# Integrated Structural, Functional, and ADMET Analysis of 2-Methoxy-4,6-Diphenylnicotinonitrile: The Convergence of X-ray Diffraction, Molecular Docking, Dynamic Simulations, and Advanced Computational Insights

**DOI:** 10.3390/molecules28196859

**Published:** 2023-09-28

**Authors:** Ahmed H. Bakheit, Hamad M. Alkahtani

**Affiliations:** Department of Pharmaceutical Chemistry, College of Pharmacy, King Saud University, P.O. Box 2457, Riyadh 11451, Saudi Arabia; ahamad@ksu.edu.sa

**Keywords:** 2-methoxy-4,6-diphenylnicotinonitrile, X-ray diffraction, orthorhombic crystal system, CH–π interaction, Hirshfeld surface, π–π stacking, density functional theory, HOMO–LUMO gap, aromaticity indices, molecular electrostatic potential

## Abstract

This study systematically investigates the molecular structure and electronic properties of 2-methoxy-4,6-diphenylnicotinonitrile, employing X-ray diffraction (XRD) and sophisticated computational methodologies. XRD findings validate the compound’s orthorhombic crystallization in the P21212 space group, composed of a pyridine core flanked by two phenyl rings. Utilizing the three-dimensional Hirshfeld surface, the research decodes the molecule’s spatial attributes, further supported by exhaustive statistical assessments. Key interactions, such as π–π stacking and H⋯X contacts, are spotlighted, underscoring their role in the crystal’s inherent stability and characteristics. Energy framework computations and density functional theory (DFT) analyses elucidate the prevailing forces in the crystal and reveal geometric optimization facets and molecular reactivity descriptors. Emphasis is given to the exploration of frontier molecular orbitals (FMOs), aromaticity, and π–π stacking capacities. The research culminates in distinguishing electron density distributions, aromatic nuances, and potential reactivity hotspots, providing a holistic view of the compound’s structural and electronic landscape. Concurrently, molecular docking investigates its interaction with the lipoprotein-associated phospholipase A2 protein. Notably, the compound showcases significant interactions with the protein’s active site. Molecular dynamics simulations reveal the compound’s influence on protein stability and flexibility. Although the molecule exhibits strong inhibitory potential against Lp-PLA2, its drug development prospects face challenges related to solubility and interactions with drug transport proteins.

## 1. Introduction

In the realm of drug discovery and design, pyridine derivatives have consistently been of interest due to their notable bioactive properties, as evidenced by several seminal studies [1,2]. Given the importance of understanding structure–reactivity relationships, our study focuses on 2-methoxy-4,6-diphenylnicotinonitrile, a distinguished member of this class of compounds. The intention behind this investigation is to elucidate its structural characteristics, paving the way for further pharmacological exploration.

Recent advancements have spotlighted the significance of nicotinonitrile (3-cyanopyridine) derivatives, primarily due to their multifaceted biological activity [3]. These derivatives have been recognized for their antibacterial [4,5], antitumor [6], anticancer [7], cardiotonic [8], antiviral (specifically against avian influenza) [9], and anticonvulsant properties [10]. Certain compounds within this category exhibit an ability to inhibit sulfide:quinone oxidoreductase, which offers protection against detrimental cardiac remodeling and heart failure [11].

Beyond their biological attributes, nicotinonitriles are also renowned for their distinctive photophysical properties [3,12,13], paving the way for their potential applications in nonlinear optics [14], liquid crystal technologies [15], fluorescent molecular switches tailored to metal ion detection [16], and organic light-emitting devices [17].

The comprehensive structural and molecular analysis of this compound holds considerable importance.

A limited number of studies have addressed this topic in the existing literature [18]. Commonly, research concerning the crystallographic analysis of 2-methoxy-4,6-diphenylnicotinonitrile predominantly employs spectroscopy techniques for structure characterization, providing an overarching structure elucidation via theoretical method analyses. Building on this foundation, our research delves deeper into these theoretical methodologies, furnishing a comprehensive and intricate study. Furthermore, we introduce supplementary applications to enhance the understanding and practical implications of the molecule’s structure.

The quest for novel and efficacious drug candidates often hinges on the exploration of unique molecular structures and their inherent properties. An integral part of this pursuit involves the detailed understanding of the molecular and crystal structures of these candidates. The compound 2-methoxy-4,6-diphenylnicotinonitrile is one such compound that has recently garnered attention in the realm of molecular research. X-ray diffraction has historically been a primary tool to examine molecular and crystal structures, offering insights into atomic arrangements and intermolecular interactions [19].

Hirshfeld surface calculations provide a more nuanced understanding of molecular characteristics and intermolecular interactions [20]. The analysis of these interactions can be instrumental in predicting physicochemical properties, solubility, and stability, all of which are vital for drug development [21]. Moreover, the application of density functional theory (DFT) in predicting the structural properties of compounds has revolutionized modern computational chemistry [22], especially the WB97XD functional, which has shown consistent accuracy in various systems [23].

The molecular landscape, reactivity, and electronic properties of a compound can provide valuable insights into its potential as a drug candidate. Given the significance of such evaluations, this study sought to explore the properties of 2-methoxy-4,6-diphenylnicotinonitrile using a combination of analytical techniques.

## 2. Results and Discussion

### 2.1. Description of the X-ray Crystal Structure

**Figure 1:**
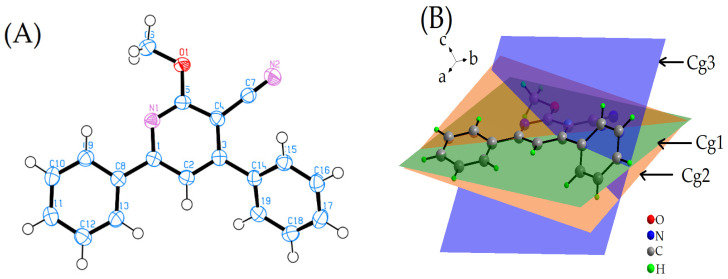
(**A**) ORTEP representation of the title compound, depicted at a 70% probability ellipsoid level, illustrating the three-dimensional shape of the electron density around the atoms. (**B**) Representation of the centroids of the rings, where Cg1 (green) incorporates atoms C1, C2, C3, C4, C5, and N1; Cg2 (brown) contains atoms C8, C9, C10, C11, C12, and C13; and Cg3 (blue) comprises atoms C14, C15, C16, C17, C18, and C19, highlighting the positioning and composition of the constituent atomic rings.

The X-ray diffraction (XRD) results confirm that the title compound crystallizes in the orthorhombic crystal system, adhering to the P21212 space group (Table 1). The asymmetric unit is composed of crystallographically independent tested molecules, as depicted in Figure 1A with ORTEP diagrams drawn at a 70% probability ellipsoid level.

The molecular structure of the 2-methoxy-4,6-diphenylnicotinonitrile molecule comprises a pyridine ring [Cg1: C1 to C5;N1], a phenyl ring [Cg2: C8–C13], and a second phenyl ring [Cg3: C14–C19]. These rings are bridged by a C1–C8 bond and a C3–C14 bond, respectively. The dihedral angle, which measures the angle between two planes, between the mean planes of the pyridine ring (Cg1) and the first phenyl ring (Cg2) is found to be 10.853(58)°. This suggests that these two rings are almost coplanar, indicating a relatively flat structure in this region of the molecule. On the other hand, the dihedral angle between the mean planes of the pyridine ring (Cg1) and the second phenyl ring (Cg3) is larger, at 42.019(53)°. This suggests a more pronounced spatial separation between these two rings, indicating a deviation from planarity and resulting in a more three-dimensional structure (Figure 1A,B) [24].

**Figure 2:**
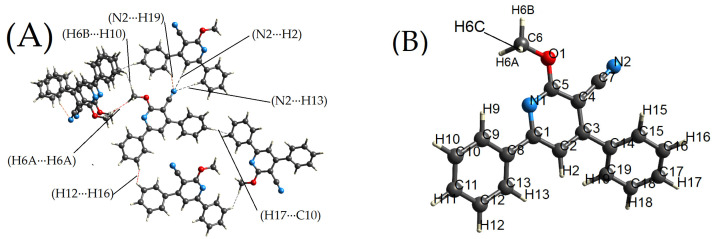
Structural illustrations of the title molecule (**A**) highlighting weak and hydrogen bonding interactions, with bonds distinctly labeled, and (**B**) detailed labeling of the molecular constituents of the title molecule.

Table 2 and Figure 2A,B describe the significant intermolecular interactions that contribute to the crystal packing of 2-methoxy-4,6-diphenylnicotinonitrile in the solid state. These interactions are significant as they determine the arrangement of the molecules in the crystal lattice and thus have a profound influence on the physical and chemical properties of the solid material. The intermolecular interactions of the title compound were found to be weak but significant. The H6B⋯H10 interaction, involving two hydrogen atoms, has an interaction length of 2.364 Angstroms, which is 0.036 Angstroms shorter than the sum of the van der Waals radii of two hydrogen atoms. This indicates a weak yet significant interaction between these atoms, contributing to the stability of the crystal structure. The H6A⋯H6A interaction also involves two hydrogen atoms. The interaction length is slightly longer (2.372 Angstroms), and the deviation from the sum of the van der Waals radii of the atoms involved is also smaller (0.028 Angstroms). This suggests that this interaction might be slightly weaker than the H6B⋯H10 interaction. The H17⋯C10 interaction, a CH–π interaction between a hydrogen atom and a carbon atom in a π system, has a length of 2.824 Angstroms and a deviation from the sum of the van der Waals radii of −0.076 Angstroms; this interaction is weaker than the hydrogen interactions but still contributes to the overall crystal packing. The interactions involving nitrogen and hydrogen atoms, namely N2⋯H19, N2⋯H2, and N2⋯H13, are likely weak hydrogen bonding or dipole–dipole interactions between the nitrogen and hydrogen atoms. Similarly, the H12⋯H16 interaction, also involving two hydrogen atoms, is analogous to the H6B⋯H10 and H6A⋯H6A interactions. While these interactions are weaker compared to strong covalent bonds, they play a crucial role in determining the crystal structure.

### 2.2. Hirshfeld Surface Calculations

The three-dimensional Hirshfeld surfaces, characterized by properties such as dnorm, di, de, curvedness, and shape index, were analyzed at a high standard resolution with an isovalue of 0.5. The molecule exhibits a surface volume of 350.32 Å^3^ and an area of 329.44 Å^2^. The globularity value of 0.729 indicates a moderately spherical structure, while the a sphericity value of 0.189 suggests that the molecule is not perfectly spherical, pointing to a degree of asymmetry in its shape [25,26].

These metrics provide a quantitative evaluation of the molecule’s spatial characteristics. Moving forward, we will explore the unique features identified within the crystalline and molecular structure of the title compound, delving into each of these properties in detail.

An extensive statistical examination of the Hirshfeld surface characteristics for a particular chemical system is shown in Table 3. The metrics comprise the normalized distance, the shape, and the curvature of the surface, as well as the distances from the Hirshfeld surface to the closest atoms inside and outside the molecule. The accompanying Sigma values shed light on the molecular interactions that shape the Hirshfeld surface and provide additional insights into the diversity of these features. Additionally, the ‘Nu’ measure is introduced, necessitating more background information for a complete understanding.

#### 2.2.1. Dnorm

**Figure 3:**
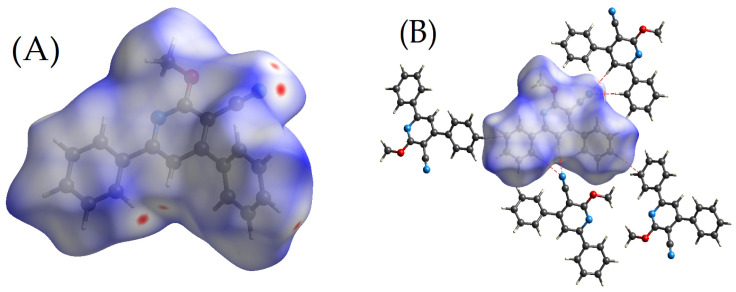
The three-dimensional Hirshfeld surface map is exhibited, with dnorm values mapped onto the surface using a red-white-blue color scheme. In this scheme, red regions signify closer contacts and a negative dnorm value, indicative of the existence of weak intermolecular interactions of various types (refer to Table 2 for details), as visible in the circular red spot within the contour in panel (**A**). White areas denote contacts around the van der Waals separation, and blue regions represent longer contacts with a positive dnorm value in panel (**A**). Panel (**B**) showcases the Hirshfeld surface, again mapped on dnorm, but, in this instance, it illustrates the connections between different molecules within the crystal via diverse types of bonds (details of these bonds can be found in Table 2).

The Hirshfeld surface, an essential tool in understanding the molecular and crystal structures of compounds, is mapped with respect to the dnorm property for the compound 2-methoxy-4,6-diphenylnicotinonitrile. This mapping employs a distinct color scheme where the color red corresponds to a value of −0.0678 (a.u), indicating a low-electron-density area, while the color blue represents a value of 1.4149 (a.u), signifying a high-electron-density region. A striking feature of this mapping is a conspicuous red spot visible within the contour of the Hirshfeld surface, as depicted in Figure 3A,B. This small red spot indicates the presence of weak intermolecular interactions of several types within the crystal structure of the compound [27]. These interactions, while weak, play a critical role in the overall structure and stability of the crystal, influencing its chemical properties and behavior. For a comprehensive understanding of these interactions, one can refer to Table 2, which provides a detailed breakdown of each type of interaction, their frequency, their relative contributions to the total surface area, and other quantitative data. Furthermore, Figure 2B provides a graphical representation of these interactions, showing how they affect the spatial arrangement of molecules within the crystal structure. This combination of quantitative data and visual representation aids in a thorough understanding of the complex interplay of forces within the crystal structure of 2-methoxy-4,6-diphenylnicotinonitrile [28,29,30].

#### 2.2.2. De and Di

The discussion of de and di values is paramount to the exploration of the electron density distribution around molecules, facilitating a profound understanding of the molecular geometry and interactions. de signifies the distance from the nearest nucleus outside to the surface, offering a depiction of the region with the maximum likelihood of encountering an electron. In contrast, di represents the distance from the nearest nucleus inside to the surface, elucidating the extent of deviation of the electron distribution from sphericity.

**Figure 4:**
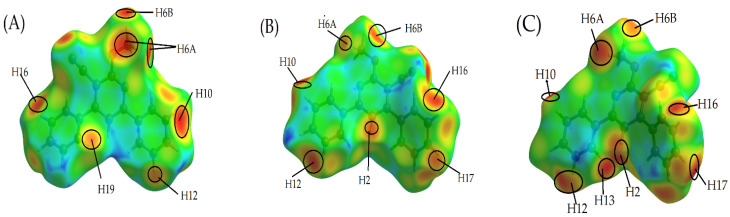
A consistent color scale was employed for both de and di conformations, using red to signify 1.0815 and 1.0820 (a.u) and blue to denote 2.4558 and 2.4371 (a.u), respectively, to optimize visual interpretation. Red spots are used to indicate the nearest nuclei relative to the surface—internally for di (**C**), and externally for de (**A**,**B**). The various hydrogen atoms involved in intermolecular interactions (refer to Table 2) serve dual roles: they are the closest internal nuclei for di and the nearest external nuclei for de, a fact visually represented by red spots within the contour.

Figure 4 presents the spatial mapping of the de and di surfaces for the molecule under consideration. The color gradient utilized is consistent for both surfaces, with red resonating with values 1.0815 and 1.0820 (a.u) and blue corresponding to 2.4558 and 2.4371 (a.u). Such visualization facilitates a clear demarcation between the de and di orientations. In the case of the titled compound, hydrogen atoms H6A, H6B, H2, H10, H12, H13, H16, H17, and H19 are situated in close proximity to the external boundary of the de surface, as showcased in Figure 4A,B. Intriguingly, these hydrogen atoms concurrently act as the proximate donor nuclei to the di surface, reflected as red contours in Figure 3C [31]. These visual inferences underscore the existence of subtle intermolecular interactions in the tested compound (refer to Table 2 for details). This postulation finds alignment with insights derived from the Hirshfeld surface analysis, mapped based on electrostatic potentials and dnorm. It is notable that both surfaces have conspicuously green, flat sections, suggesting the presence of π–π stacking interactions in the crystal packing—a paramount factor in determining molecular stability and impacting several biological functions [31].

#### 2.2.3. Curvedness

**Figure 5:**
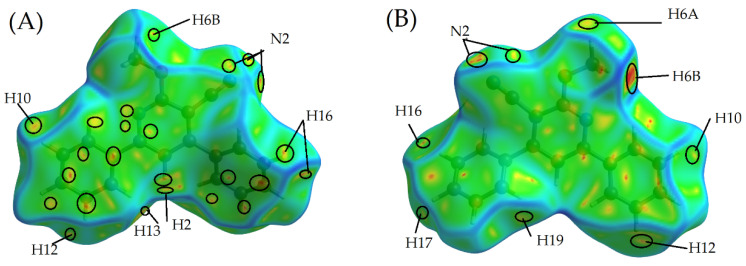
The figure displays the Hirshfeld surface, mapped according to the curvedness property, using a color scheme where −3.3 represents flat regions (depicted in red) and +0.4 indicates edges (shown in blue). Panels (**A**,**B**) provide both front and rear views of the title molecules. The presence of noteworthy flat regions over the ring structures on both sides suggests π–π stacking interactions. These interactions are represented as yellow spots located within black-colored contour lines.

The Hirshfeld surface, an insightful tool in analyzing molecular and crystal structures, is mapped in three dimensions with respect to the curvedness property for the compound 2-methoxy-4,6-diphenylnicotinonitrile. This specific molecule is characterized by a planar configuration comprising three aromatic rings. In the curvedness mapping visualized in Figure 5 [32], a color coding scheme is employed, with red (–3.3) representing flat regions of the molecule and blue (+0.4) delineating the edges. This color scheme aids in the clear differentiation of the molecular structure’s contours and provides a vivid visual representation of its three-dimensional configuration. One of the most intriguing features of this mapping is the presence of yellow to red spots within the contours, which represent areas of very weak intermolecular interactions. These interactions can be further understood by referring to Table 2, which provides a more detailed breakdown of the types and extent of these interactions. Notably, there are green-colored flat regions circumscribed by the red spots on the surface, particularly around the ring atoms (the rings on either side of the same molecule). The presence of these green regions is significant as it confirms the existence of π–π stacking interactions in the crystal packing of the title compound. π–π stacking effects, which typically occur between aromatic rings or between aromatic rings and the π electrons of double bonds, are a key consideration in the study of crystal packing.

##### Shape Index

**Figure 6:**
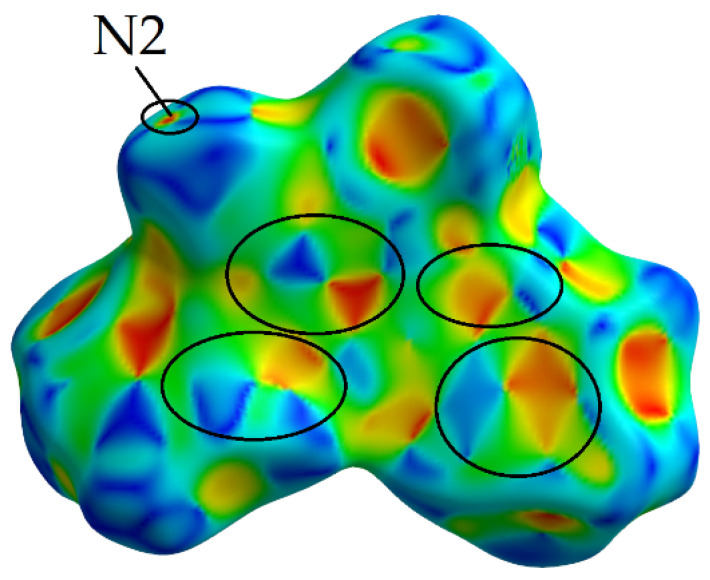
This image depicts the Hirshfeld surface, mapped according to the shape index. The presence of a red spot nestled within the contour signifies a very weak intermolecular interaction. The appearance of triangles colored in red and blue indicates the existence of π–π interactions. The blue protrusions and red indentations represent points of contact between neighboring molecular Hirshfeld surfaces.

The Hirshfeld surface, a valuable tool in visualizing and understanding the intricacies of molecular and crystal structures, has been mapped based on the shape index for this analysis. The shape index is a unique measure that provides a two-valued descriptor of the shape at each point on a surface, distinguishing between convex, concave, and flat regions. For this particular mapping, depicted in Figure 6, a color scheme has been employed where −1.0 signifies concave regions (represented in red) and +1.0 indicates convex regions (portrayed in blue). One of the distinct features of this mapping is the red spot nestled within the contour. This spot is a clear confirmation of the existence of very weak intermolecular interactions present within the structure, a fact that is further elaborated in Table 2. Despite being weak, these interactions play a crucial role in defining the overall structure and stability of the compound. In addition to the red spot, the mapping reveals several smaller regions displaying a yellowish-red hue. These concave areas are indicative of additional weak intermolecular interactions that are also present within the structure, further contributing to its stability and unique properties. A noteworthy feature of the shape index mapping of this test compound is the presence of red- and blue-colored triangles. These triangles, which represent regions of varying curvature on the Hirshfeld surface, are indicative of the existence of π–π interactions within the crystal packing of the compound, as stated in reference [20]. π–π interactions, commonly occurring between aromatic rings or between aromatic rings and the pi electrons of double bonds, are of significant importance in the crystal packing and stability of many compounds. Their presence in the crystal packing of this test compound sheds light on the compound’s specific structural characteristics and behavior.

#### 2.2.4. The Fragment Patch

The fragment patch analysis of the Hirshfeld surface for the compound 2-methoxy-4,6-diphenylnicotinonitrile provides valuable insights into the intermolecular interactions within its crystal structure; see Figure 5A–D. This is achieved by examining the area of each fragment patch, which corresponds to the closest atomic contacts on the Hirshfeld surface. From the provided data, it is observed that the fragment patches vary significantly in size, ranging from 6.0 Å^2^ to 71.7 Å^2^ (Table 4).

**Figure 7:**
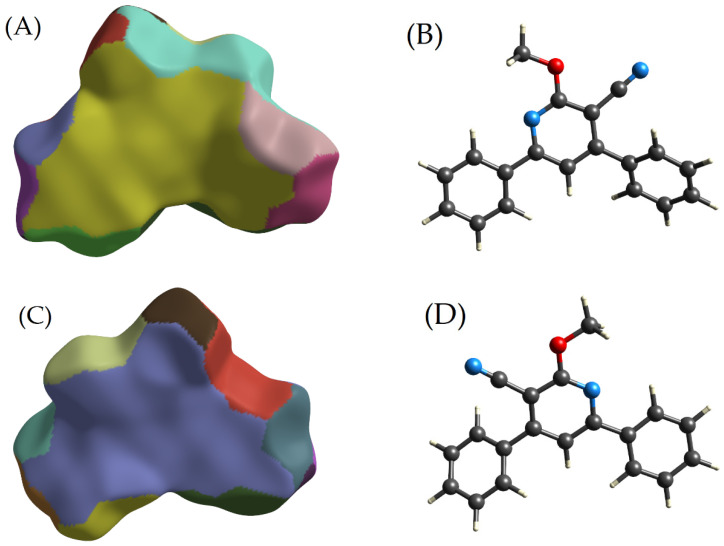
This figure displays the Hirshfeld surface, mapped in accordance with the fragment patch, featuring identifiers from 0 to 15. The presented images, labeled as (**A**–**D**), offer both front and rear views of the title molecules, with and without contours, respectively, for a comprehensive visualization. Particularly noteworthy are the substantial flat and expansive areas corresponding to fragment patches 2 and 15 located over the rings on either side of the structure, indicative of π–π stacking. Conversely, smaller fragment patch areas scattered across the surface represent weaker interactions.

This considerable variation indicates a broad range of interaction strengths present in the crystal structure of the compound. In particular, the fragment patches labeled as 2 (Figure 7A,B) and 15 (Figure 7C,D), which measure 69.9 Å^2^ and 71.7 Å^2^, respectively, stand out due to their large size. The significant expanses of these patches suggest that the atoms that they correspond to are involved in robust intermolecular interactions. These interactions contribute substantially to the overall stability and behavior of the compound. Moreover, the large areas of these patches also hint at the presence of pi–pi interactions within the crystal packing of the compound. pi–pi interactions, typically occurring between aromatic rings or between aromatic rings and the pi electrons of double bonds, are of significant importance in the crystal packing and stability of many compounds. Their presence in this compound sheds more light on its unique structural characteristics and behavior. On the other end of the spectrum, fragment patches 5, 11, and 16, with areas of 7.7 Å^2^, 6.0 Å^2^, and 8.7 Å^2^, respectively, represent the smallest patches. These smaller areas suggest that the corresponding atoms are involved in weaker intermolecular interactions within the crystal structure. The remaining fragment patches, with areas ranging from 9.1 Å^2^ to 31.7 Å^2^, indicate a range of intermediate-strength interactions. These interactions could involve a variety of bonds, such as van der Waals forces, dipole–dipole interactions, or weaker hydrogen bonds.

#### 2.2.5. Two-Dimensional Fingerprint Plots

**Figure 8:**
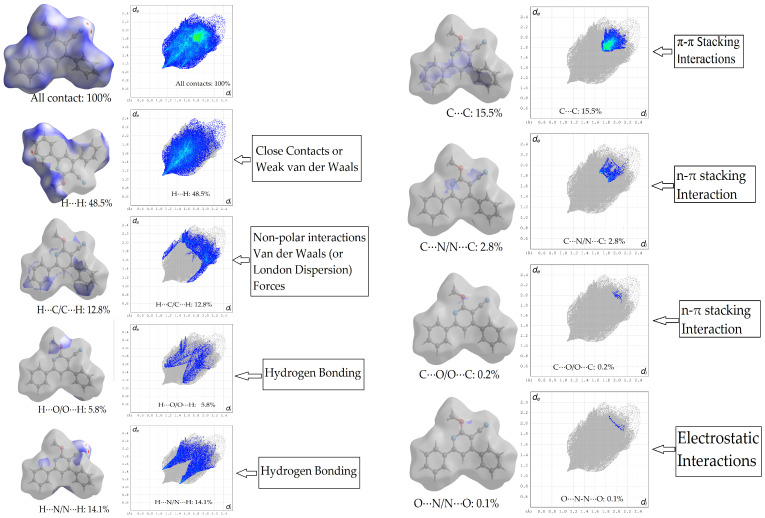
This illustration showcases the 2D and 3D fingerprint plots of the test compound. In the 2D plot, the *y*-axis denotes ‘de’ (distance to the nearest external element) and the *x*-axis shows ‘di’ (distance to the nearest internal element) of the 3D Hirshfeld surface. Blue spots represent specific X⋯Y element pair contributions, while grey spots outline the combined contributions of all interacting pairs. The left depicts dnorm surface patches and their contribution percentages, while the right highlights intramolecular interactions with neighboring molecules in the compound crystal.

The two-dimensional fingerprint plots serve as a comprehensive visual depiction of the intermolecular interactions at play within the crystal structure. These plots also quantify the specific contributions of each interaction type towards establishing the three-dimensional Hirshfeld surface, thereby providing a detailed portrayal of the compound’s molecular dynamics. In our investigation, we have derived two-dimensional fingerprint plots for all possible pairs of contact points (as shown in Figure 7 and Figure 8). An extensive analysis of these plots yielded specific results, which are catalogued systematically in Table 5.

**Figure 9:**
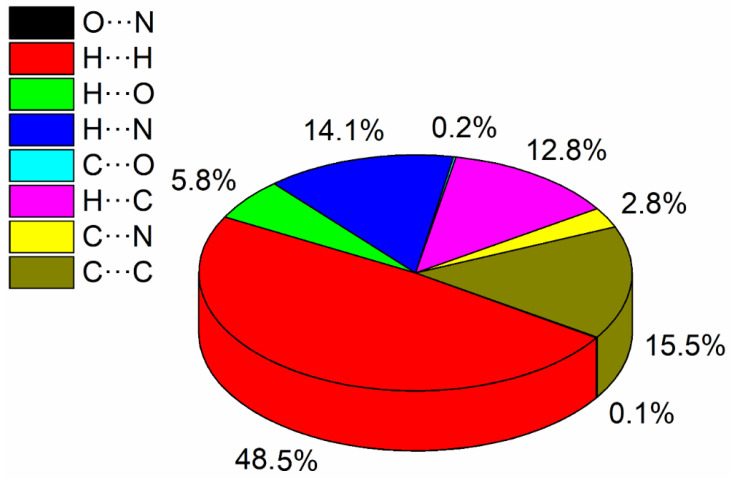
The figure delineates the relative contributions of diverse intermolecular contacts to the Hirshfeld surface area in the title compound, each represented by a unique color for clear differentiation, accompanied by a corresponding key.

Based on the presented Figure 8 and Figure 9, it can be inferred that the H⋯H interaction holds a prominent position, accounting for 48.5% of the overall interaction strength. This observation implies that weak van der Waals forces exert a strong influence on the cohesive nature of the compound’s crystal structure. The prevalence of this interaction at a significant proportion can exert influences on the properties of the crystal, potentially resulting in reduced density, increased malleability, and a potentially lower melting point in comparison to crystals that are predominantly governed by stronger intermolecular forces.

According to the results from the fingerprint plot analysis and the interpretation of the intermolecular interactions in the crystal packing of the test compound in Table 2, the interactions between hydrogen and oxygen atoms, denoted as H⋯O, contribute to around 5.8% of the overall molecular forces. However, contrary to what is commonly observed, these interactions are not strong hydrogen bonds. Instead, they are weak intermolecular interactions, less potent than hydrogen bonds but still contributing stability to the crystal structure. These weak H⋯O interactions arise from the electrostatic attraction between hydrogen and the more electronegative oxygen atoms, influencing the physical properties of the crystal, including characteristics such as melting and boiling points.

Similarly, the hydrogen–nitrogen (H⋯N) interactions, accounting for approximately 14.1% of the total intermolecular forces, are not the strong hydrogen bonds often seen in biological structures. Instead, these are weaker intermolecular forces, resonating with the overall weak interaction theme seen in the crystal packing of the investigated compound. Despite their relative weakness, these H⋯N interactions play a significant role in stabilizing and defining the shape of the crystal structure. The interactions between hydrogen and carbon atoms, denoted as H⋯C interactions, account for approximately 12.8% of the overall contribution to the stability of the crystal structure. These interactions are commonly associated with van der Waals forces, which, although weaker than hydrogen bonds, still play a significant role in maintaining the structural integrity of the crystal.

The carbon–carbon (C⋯C) interaction within the crystal structure, contributing 15.5% according to the provided data, plays a pivotal role in maintaining the stability and influencing the properties of the crystal structure. Given that this structure includes aromatic rings, a significant portion of these C⋯C interactions are likely due to π–π stacking. These π–π stacking interactions, arising from overlapping p-orbitals in adjacent aromatic rings, can significantly affect the way in which the molecules pack within the crystal structure, often leading to a more closely packed and stable arrangement. Additionally, these interactions may have a notable impact on other properties of the crystal, such as its light absorption and emission characteristics, which are an important consideration in the area of optical materials. In brief, the H⋯H contact holds primary importance inside the crystal structure, whereas the remaining interactions also exert a substantial influence on the crystal’s characteristics and stability. Gaining a comprehensive understanding of the intricate dynamics of these interactions might yield useful insights into the behavior of the material across various conditions.

### 2.3. Energy Frameworks

The energy framework is an essential tool used to comprehend the various energy forms that play a role in the supramolecular assembly of molecules within a crystal [33]. The energy framework calculations in this study were conducted using the CrystalExplorer 17 software, which is widely acknowledged as a reliable tool for this specific investigation. The computations utilized a B3LYP/6-31G(d,p) functional basis set, which is widely recognized and employed in the field of computational chemistry. The calculation of interaction energies was performed for a cluster with a radius of 3.8 Å centered around a solitary molecule of the substance under investigation. The selected scale factors aligned with previously published values [29,30,33].

**Figure 10:**
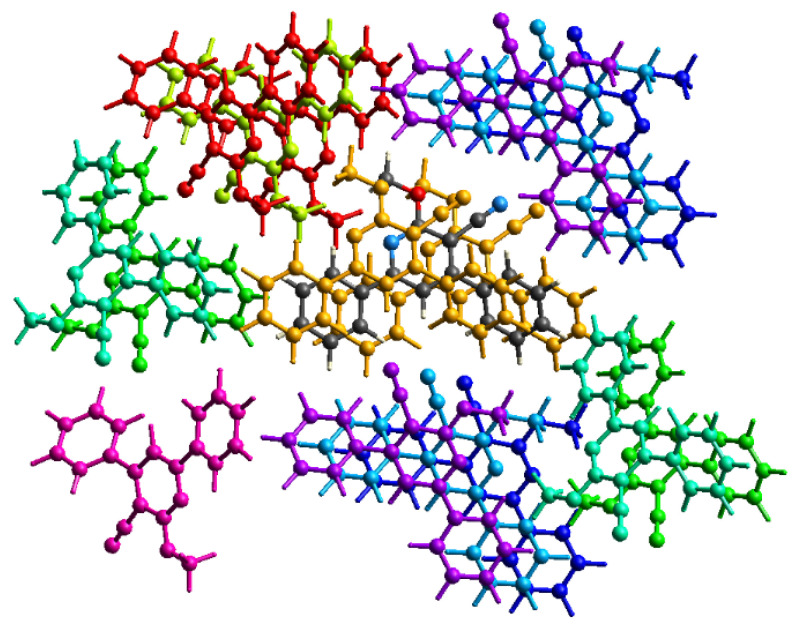
The molecular interactions involving the central molecule and the surrounding molecules forming a cluster with a radius of 3.8 Å.

**Figure 11:**
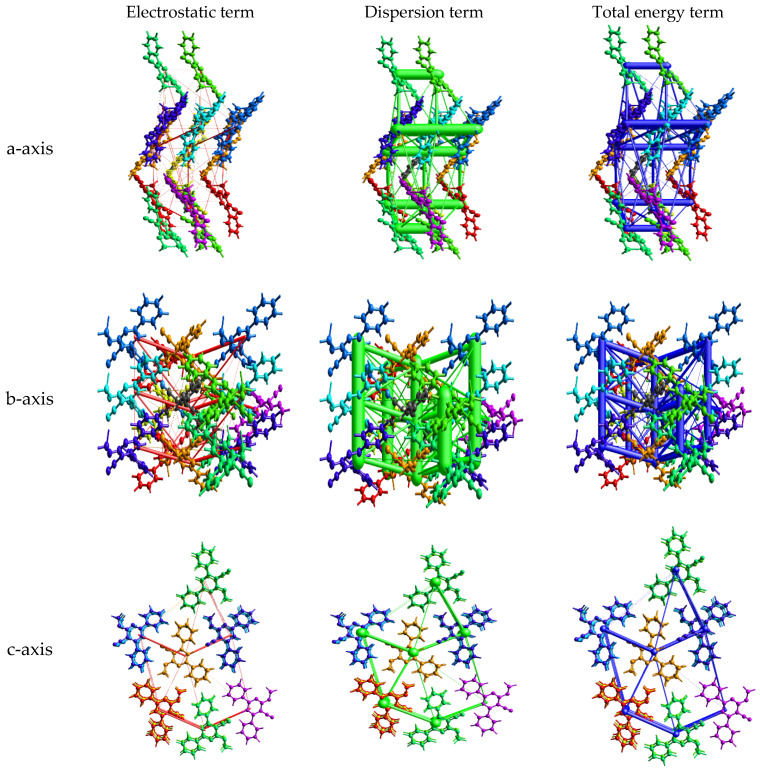
This figure presents the energy components for the examined compound, as viewed along the crystallographic a, b, and c axes. The thickness of the solid cylinders within the framework corresponds to the magnitude of the interaction energies between pairs of molecules. In the total energy frameworks, the molecules represented in orange and blue denote the highest interaction energy, which is −54.3109 kJ/mol, as detailed in Table 6 and Table 7.

The three-dimensional energy frameworks in Figure 10 and Table 6 illustrate the molecules surrounding the central molecule (depicted by a black ball and stick model) within a default radius of 3.8 Å. These molecules are color-coded for clarity. The energy frameworks for the primary compound were developed using default red-, green-, and blue-colored solid cylinders, representing Coulombic or classical electrostatic energy (E_ele_), dispersion energy (E_dis_), and total energy components (E_tot_), respectively. These frameworks, as displayed in Figure 10, are visualized along the crystallographic a, b, and c axes for the molecules under examination. Upon detailed examination of Figure 10 and Figure 11, one can notice that the cylinder representing the dispersion energy is noticeably thicker than the cylinders corresponding to the other energy types. The larger size of the dispersion energy cylinder is indicative of the substantial contribution of dispersion intermolecular interactions to the energy framework of the crystal.

The varying thickness of the solid cylinders in each energy framework signifies the relative interaction strength between their constituent molecular units, which is further substantiated by the noticeably higher negative energy values (refer to Table 4).

Furthermore, these tables also detail a color-coding scheme for molecules interacting at different Cartesian coordinates, and they provide information about the number of interacting pairs with the central molecule (N), their molecular centroid distance (R), and the rotational symmetry operation (symmetry). These details are crucial in calculating the lattice energy of a crystal. The interaction energy calculations within the molecule’s cluster indicate that the highest total interaction energy (E_tot_ − 54.31 kJ/mol) is linked to two molecules (signified by an orange color in Figure 9, Figure 10 and Figure 11). These molecules, with the symmetry operation (x, y, z), are positioned above and below the plane of the primary molecule at a molecular centroid distance (R) of 3.9 Å, resulting from π–π stacking interactions (Figure 11). The second highest total interaction energy (−27.85 kJ/mol) is associated with two molecules (depicted by a sky color in Figure 9, Figure 10 and Figure 11). These molecules, with the symmetry operation (x + 1/2, −y + 1/2, −z), are located at the sides of the molecule at a distance of 8.28 Å (Figure 11). They result from the weak intermolecular interactions of N2⋯H2–C2 and N2⋯H13–C13.

The weakest interaction is with two violet–colored molecules at a molecular centroid distance (R) of 14.38 Å and with the symmetry operation (−x, −y, z), indicating the lowest total interaction energy (−1.22 kJ/mol). As anticipated, in the molecule’s cluster, the total interaction energies are weaker for molecules at longer centroid distances, aligning with the principles of electrostatics.

Additionally, the conversion factors (kele, kdis, kpol, and krep) for total energies are only reported for two benchmarked energy models of electron density functions (CE-HF…HF/3-21G and CE-B3LYP … B3LYP/6-31G(d,p)), and these are appropriately scaled (shown under Table 4). From the overall study of energy frameworks, it emerges that the dispersion energy (E_dis_) components dominate the classical electrostatic energy (E_ele_) frameworks (Figure 11).

The 2-methoxy-4,6-diphenylnicotinonitrile molecule, in its crystalline form, boasts a total interaction energy of −141.43 kJ mol^−1^, a cumulative product of various energy contributions. Among these contributions is the electrostatic energy, amounting to −29.07 kJ mol^−1^, which signifies the energy associated with the interaction of the molecule’s charged components, with a negative value indicating the presence of attractive forces. Polarization energy, at −16.04 kJ mol^−1^, corresponds to the molecule’s ability to modify its electron cloud in response to the nearby presence of other molecules. Dispersion energy, alternatively known as van der Waals or London dispersion forces, is set at −191.54 kJ mol^−1^ and represents the energy resulting from temporary correlations within the electron movements of the interacting molecules. Lastly, the repulsion energy, amounting to 110.05 kJ mol^−1^, is indicative of the repulsive forces that arise when the electron clouds of the interacting molecules come into close proximity, as demonstrated by its positive value. In the 2-methoxy-4,6-diphenylnicotinonitrile crystal, the most potent force is the dispersion energy, given its magnitude of −191.54 kJ mol^−1^, which indicates that the transient correlations in electron movement between interacting molecules play a crucial role in the overall stability of the crystal structure (Figure 11).

In a study by Mackenzie et al. [33], scale factors corresponding to the data in Table 6 were presented for two benchmarked energy models. For the CE-HF model utilizing HF/3-21G electron densities, the assigned scale factors were k_ele = 1.019, k_pol = 0.651, k_disp = 0.901, and k_rep = 0.811. Similarly, for the CE-B3LYP model incorporating B3LYP/6-31G(d,p) electron densities, the scale factors were k_ele = 1.057, k_pol = 0.740, k_disp = 0.871, and k_rep = 0.618. These factors play a crucial role in adjusting the components of interaction energy in computational chemistry models.

**Figure 12:**
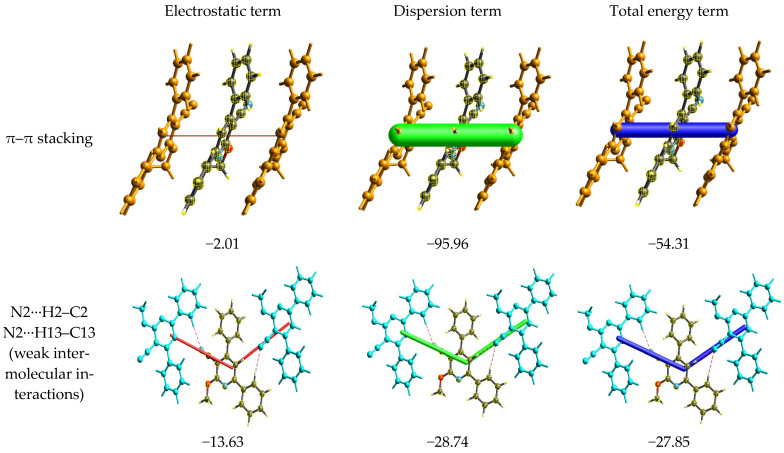
The figure presents interaction energies for the title compound as calculated using the CE-B3LYP model. Evident from the interaction energies table, the dominant interaction occurs through the π–π stacking motif between the central molecule (highlighted in yellow mesh) and its x, y, z symmetry-related molecule (depicted in orange), yielding an interaction energy of −54.3109 kJ mol^−1^. Subsequently, the second most robust interaction is characterized by an energy of −27.85 kJ mol^−1^, involving the N2⋯H2–C2, N2⋯H13–C13 motifs (indicative of weak intermolecular interactions) between the central molecule and the x + 1/2, −y + 1/2, −z symmetry-related molecule (represented in dodger blue). The cylinders are color-coded as red, green, and blue, signifying the electrostatic term, dispersion term, and total energy term, respectively. Additionally, the thickness of the solid cylinders corresponds to the magnitude of interaction energies between molecular pairs.

Based on the provided results in Figure 12, two primary interactions are presented: π–π stacking and weak intermolecular interactions involving N2 with H2–C2 and H13–C13. Analyzing the energy contributions, we can observe the following.

1. For the π–π stacking interaction, the electrostatic term is −2.01, while the dispersion term significantly contributes with a value of −95.96. The combination of both these interactions leads to a substantial total energy term of −54.31. This suggests that the π–π stacking interaction is predominantly stabilized by the dispersion forces, which are known to be critical for π systems. While the electrostatic contribution is minimal in this context, it still plays a role in determining the overall energy of the interaction.
2. For the weak intermolecular interactions involving N2, the electrostatic contribution is −13.63, which is notably higher in magnitude than that observed for π–π stacking. The dispersion term for this interaction is −28.74, which, while still significant, is considerably less dominant than its counterpart in the π–π stacking. The total energy term for this interaction is −27.85, indicating that both electrostatic and dispersion interactions are nearly equally contributing to the stability of these weak intermolecular contacts.

In summary, while the π–π stacking is largely governed by dispersion forces, the weak intermolecular interactions are more balanced in their energy contributions, with both electrostatic and dispersion terms playing important roles.

### 2.4. Density Functional Theory (DFT) Computations

#### 2.4.1. Optimization of the Structure

**Figure 13:**
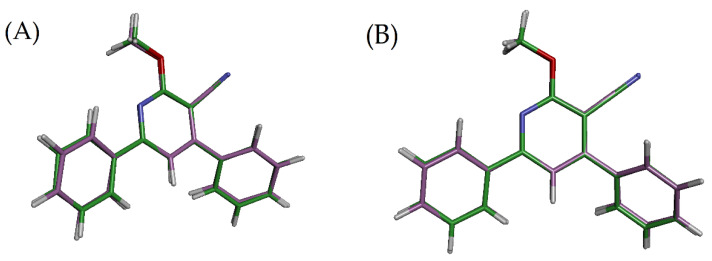
Overlapping of the X-ray crystal structure with the optimized structure of the compound. (**A**) The superimposed structures using the [WB97XD/6-311G(d,p)] optimized basis set, where the X-ray crystal structure is depicted in green and the theoretical structure in violet. (**B**) The aligned structures employing the [B3LYP/6-311G(d,p)] optimized basis set, with the X-ray crystal structure again illustrated in green and the theoretical structure in violet.

The geometric optimization of the molecular structure of the compound was conducted at two different theory levels, DFT/B3PW91/6-311G(d,p), DFT/WB97XD/6-311G(d,p), and DFT/B3LYP/6-311G(d,p). These optimized structures are visually presented in Figure 13. The bond lengths and angles calculated from all theoretical levels were then juxtaposed with the experimental data derived from X-ray diffraction, as documented in Table 7. An in-depth examination of Table 7 reveals a compelling comparison of the experimental and theoretical bond lengths. Impressively, the study shows stronger alignment between the experimental values and the calculations made with DFT/B3PW91/6-311G(d,p) and DFT/WB97XD/6-311G(d,p) than with DFT/B3LYP/6-311G(d,p). This finding underscores the superior reliability of the DFT/B3PW91/6-311G(d,p) and WB97XD function over the B3LYP in these particular theoretical calculations. The reliability of the B3PW91 and WB97XD function is further substantiated by the mean absolute error (MAE) calculations. The MAE for B3PW91 and WB97XD is noted to be 0.004825 and 0.0065375, which is significantly lower than the MAE for B3LYP, recorded as 0.0220125. This result suggests that the B3PW91 and WB97XD methods provide a more accurate prediction of the molecular geometry, reflecting a closer correspondence with the experimental data. Therefore, for theoretical calculations involving this particular compound, the B3PW91 and WB97XD function appears to be the more reliable choice [34].

Table 8 presents a detailed comparison between the experimental bond angles and those obtained from the molecular structure’s geometric optimization. The correlation between the experimental data and the values produced by the WB97XD and B3PW91 functions is notably stronger than with the B3LYP function. This observation is solidified by the mean absolute error (MAE) values: 0.386275° for WB97XD, 0.42631875° for B3PW91, and a notably higher 1.62583125° for B3LYP. A smaller MAE signifies that the theoretical prediction closely mirrors the experimental data. Consequently, the considerably reduced MAE for WB97XD and B3PW91 indicates that their predictions align better with the experimental observations. Therefore, the WB97XD and B3PW91 functions are more reliable than the B3LYP in predicting this compound’s molecular structure’s bond angles, making them preferable for theoretical geometric optimization.

#### 2.4.2. Global Reactivity Descriptors for the Investigated Compound

The analysis of the global reactivity descriptors for 2-methoxy-4,6-diphenylnicotinonitrile provides insightful information about its chemical reactivity and stability, as well as its potential interactions with other compounds. These indices were calculated through conceptual density functional theory (CDFT), which provides a theoretical framework for an understanding of the electronic structures of molecular systems. As shown in Table 9, the vertical ionization potential (IP) of the compound is 7.7691 eV. This value quantifies the minimum energy required to remove an electron from the molecule, indicating the stability of the compound towards oxidation. A high IP generally suggests a lower tendency to lose an electron and, hence, lower reactivity. The vertical electron affinity (EA) of the compound, which measures the energy change when an additional electron is added to the molecule, is relatively low (0.3931 eV). This suggests that the compound is not particularly prone to reduction. The Mulliken electronegativity of the molecule is 4.0811 eV. Electronegativity is a measure of the ability of an atom or molecule to attract electrons. Higher electronegativity suggests a stronger ability to attract electrons. The chemical potential is −4.0811 eV, which is equivalent to the negative of the Mulliken electronegativity. This descriptor reflects the tendency of the molecule to accept or donate electrons. A lower (more negative) chemical potential suggests higher reactivity. The calculated hardness of the molecule is 7.3759 eV; this measures the resistance of the molecule to changes in electron configuration. Higher hardness indicates greater stability and lower reactivity. In contrast, the softness, which is the reciprocal of the hardness, is 0.1356 eV^−1^. Higher softness suggests higher reactivity. The electrophilicity index is 1.1290 eV; it is a measure of the propensity of the molecule to accept electrons (i.e., to act as an electrophile). A higher electrophilicity index indicates the higher likelihood of the molecule to be attacked by nucleophiles. Finally, the nucleophilicity index is 2.8345 eV. This descriptor provides an indication of the ability of the molecule to donate electrons (i.e., to act as a nucleophile). A higher nucleophilicity index suggests a greater tendency for the molecule to attack electrophiles [35,36,37].

#### 2.4.3. Analysis of Local Reactivity Indices for the Examined Molecule

The local reactivity descriptors, specifically the electrophilic $P_{k}^{+}$ and nucleophilic $P_{k}^{-}$ Parr functions, provide valuable insights into the reactivity behavior at the atomic level for the 2-methoxy-4,6-diphenylnicotinonitrile compound. These functions offer a detailed understanding of how each atom within the compound behaves in terms of electrophilic and nucleophilic reactivity.

**Figure 14:**
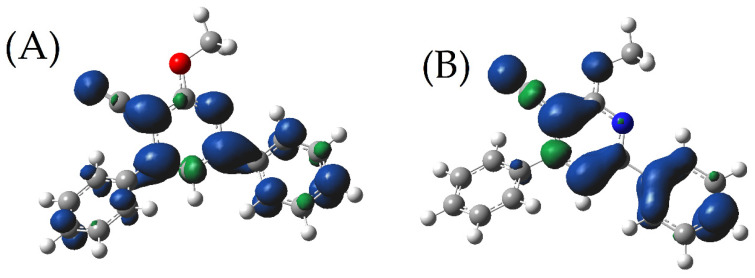
Illustrations of Mulliken atomic spin densities for radical anions (**A**) and radical cations (**B**), together with nucleophilic and electrophilic Parr functions for the analyzed molecule. Positive spin densities are denoted in blue, while negative ones are shown in green.

The electrophilic Parr function $P_{k}^{+}$ measures the propensity of an atom to accept an electron pair (i.e., act as an electrophile). From the provided data, C4 has the highest electrophilic Parr function value (9.618), indicating that this carbon atom is the most likely to accept an electron pair, thereby making it the most electrophilic site within the compound; see Figure 14A.

On the other hand, the nucleophilic Parr function $P_{k}^{-}$ provides an indication of the tendency of an atom to donate an electron pair (i.e., act as a nucleophile). In this case, C1 has the highest nucleophilic Parr function value (6.829), suggesting that this carbon atom is the most likely site for electron pair donation, making it the most nucleophilic site within the molecule; see Figure 14B.

Interestingly, some atoms show a significant difference between their electrophilic and nucleophilic tendencies. For instance, C2 has a high electrophilic value (5.788) and a negative nucleophilic value (−2.458), demonstrating that this atom is more prone to accepting electrons rather than donating them. Conversely, C3 shows a negative electrophilic value (−3.521) and a high nucleophilic value (5.293), suggesting that this atom is more likely to donate electrons rather than accept them. Overall, these local reactivity descriptors allow us to identify the most reactive sites within the 2-methoxy-4,6-diphenylnicotinonitrile compound and understand their behavior in potential chemical reactions. This information can be particularly useful in predicting the compound’s interactions with other molecules and its behavior in various chemical environments (Table 10).

#### 2.4.4. Frontier Molecular Orbitals (FMOs)

**Figure 15:**
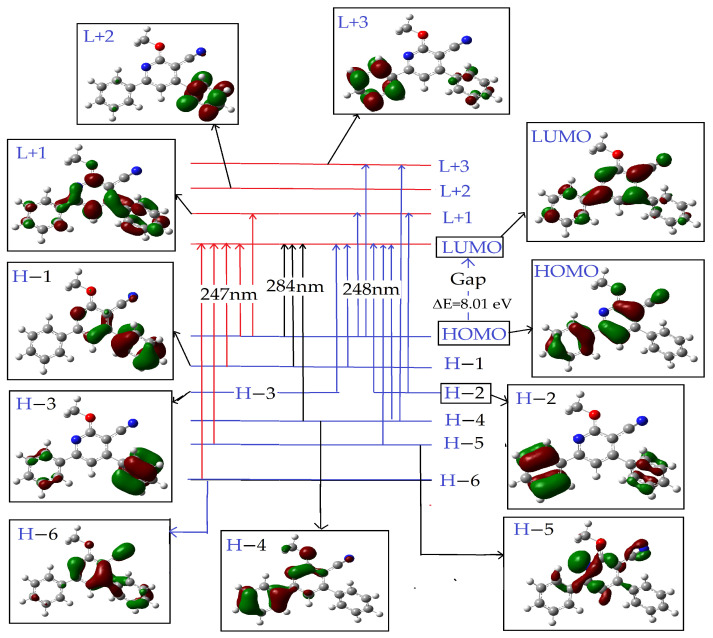
Visualization of the frontier molecular orbitals for the specified molecule, calculated using the DFT methodology with the wB97XD functional and the 6-311G(d,p) basis set under gas-phase conditions. Red color indicates positive electron density, while green color signifies negative electron density. This display encompasses the highest occupied molecular orbital (HOMO) and its lower-energy counterparts H–1, H–2, H–3, H–4, H–5, and H–6, as well as the lowest unoccupied molecular orbital (LUMO) and its higher-energy counterparts L+1, L+2, and L+3. These visual representations also highlight the major and minor contributions of atomic orbitals, facilitating a better understanding of possible electronic transitions and interactions within the molecule.

The frontier molecular orbitals (FMOs), specifically the highest occupied molecular orbital (HOMO) and the lowest unoccupied molecular orbital (LUMO), are critical in assessing a molecule’s chemical stability and electronic properties. These orbitals serve as key determinants in molecular interactions with other chemical entities, providing insights into the molecule’s reactivity and interaction potential. Figure 15 offers a detailed visualization of these FMOs, enabling an in-depth exploration of the compound’s bonding schemes and potential for electronic transitions.

The frontier molecular orbitals offer profound insights into the electronic behavior and interactions of the specified molecule; see Table 11 and Figure 15. The first transition, occurring at a wavelength of 283.9 nm and corresponding to an energy of 35227.9 cm^−1^, boasts a significant oscillator strength of 0.5084. This suggests a high probability of this transition. The dominant pathway for this transition is from the HOMO to the LUMO, contributing 86% of the entire transition. Notably, the other minor contributions come from the H–4 to LUMO and H–1 to LUMO transitions, contributing 2% and 4%, respectively. This pronounced HOMO->LUMO transition emphasizes the molecule’s π–π* interaction, a signature of conjugated systems.

The second transition, identified at an energy of 40,377.7 cm^−1^ and a wavelength of 247.7 nm, has a modest oscillator strength of 0.0355. Predominantly, this transition is governed by the H–5 to LUMO and H–2 to LUMO pathways, contributing 38% and 19%, respectively. Several minor pathways contribute to this transition, with particular emphasis on the H–4 to LUMO and H-4 to L+3 transitions. These diverse contributors signal varied electronic interactions within the molecule. The complex interplay suggests potential overlaps between different orbital types, pointing towards mixed σ–π and π–π* interactions.

Transition three, discerned at 247.1 nm or 40,469.7 cm^−1^, has an oscillator strength of 0.0694. This transition prominently involves the H–5 to LUMO and H–1 to LUMO pathways, with impressive contributions of 27% and 50%, respectively. Additionally, there are minor contributions, notably the H–6 to LUMO and HOMO to L+1 transitions. The significant presence of the H–1 to LUMO transition within this energy suggests extensive electron transfer involving the molecular region associated with the H–1 orbital.

Collectively, these transitions and their contributing orbitals provide an intricate picture of the electronic structure of the molecule. The most dominant HOMO->LUMO transition speaks to the molecule’s reactivity and its potential for π–π interactions. Meanwhile, the varied minor contributors in the following transitions illuminate the complex electronic landscape of the molecule, indicative of a spectrum of bond types and molecular orientations.

The energy differential between these orbitals, commonly referred to as the HOMO–LUMO gap, offers insights into the molecular stability, reactivity, and electronic properties of a compound [38]. A smaller HOMO–LUMO gap often indicates increased electrical conductivity and diminished kinetic stability. The HOMO represents the molecule’s capacity to donate electrons, while the LUMO signifies its ability to accept electrons [39]. For the molecule under investigation, 75 out of the 480 molecular orbitals are occupied. The presented Table 11 provides a detailed analysis of the molecular orbitals (MOs) of the compound under investigation, specifically focusing on the distribution of electron density between the phenyl group (C6H5) and 2-methoxy-nicotinonitrile (C7H3N2O) components of the molecule. Each row of the table represents a specific molecular orbital, characterized by its energy level (eV), symmetry (A), and the percentage contribution of each component to the orbital. It also includes accurate values for the distribution of electron density. Notably, the HOMO (75th orbital) and LUMO (76th orbital) are highlighted, with energy levels of −8.41 eV and −0.4 eV, respectively. The electron density in the HOMO is majorly contributed by the C7H3N2O component (66%), while, in the LUMO, it is reversed, with C7H3N2O contributing 69%.

**Figure 16:**
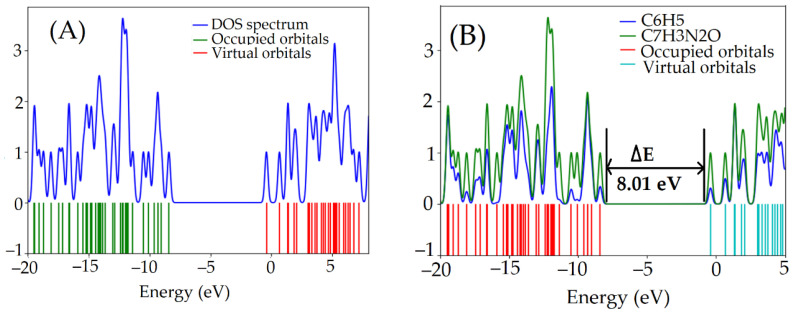
The computational results for the molecule under study, with (**A**) showcasing the total electronic density of states and (**B**) displaying the partial density of electronic states.

**Figure 17:**
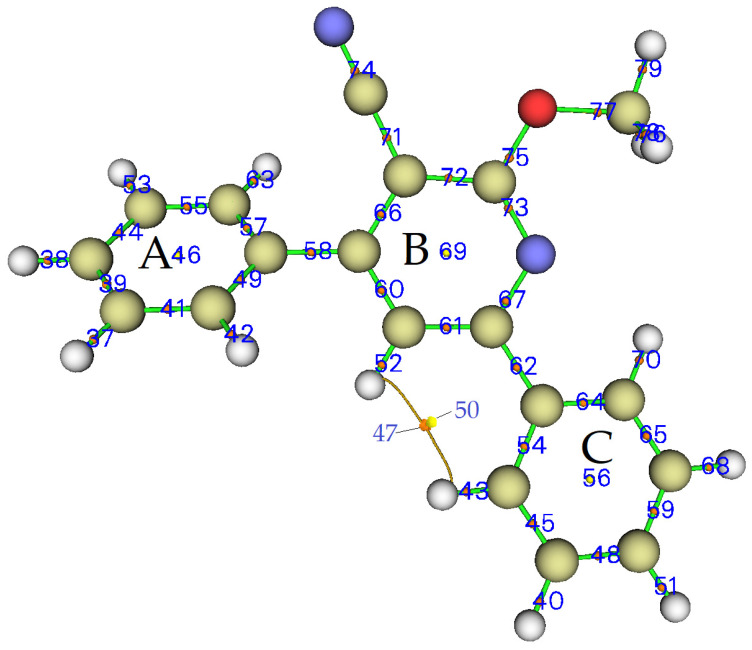
The figure presents a topology analysis for electron density on the title compound, optimized at the WB97XD/6-311G(d,p) level of theory using the Multifunctional Wavefunction Analyser (Multiwfn). The molecular structures illustrated include labels for rings and pseudorings, with the phenyl rings represented by (A) and (C), and the six-membered ring of diphenylnicotinonitrile designated as (B). Orange spheres represent critical points for H-bonds, while yellow spheres correspond to those for rings. Bond paths are denoted by brown lines, and the indices of Critical Points (CPs) are labeled with blue numbers. These detailed renderings were generated using MULTIWFN.

The density of states (DOS) spectrum provides a comprehensive analysis of the energy levels within a specified energy range, (ΔE), as illustrated in Figure 16. When this spectrum is integrated with contributions from distinct atomic or functional groups, it is referred to as the partial density of states (PDOS), as delineated in Figure 15 and Figure 16 and Table 12 [40]. Such data facilitate the identification of the electronic contributions of diverse molecular fragments to both the highest occupied molecular orbital (HOMO) and the lowest unoccupied molecular orbital (LUMO). Evidenced by Figure 16B and Figure 17, the HOMO, anchored at −8.41 eV, predominantly garners its attributes from the phenyl (C6H5) segment (r-ring (C), refer to Section 2.4.5.) and the 2-methoxy-nicotinonitrile (C7H3N2O) fragment, contributing 34% and 66%, respectively. Such dominance underscores the principal role that these groups play as electronic state occupiers, hinting at their potential electron-donating capacity in chemical interplays. In contrast, Figure 15 and Figure 16 and Table 12 elucidate that the LUMO, situated at −0.4 eV, predominantly derives its characteristics from the same HOMO fragments, with contributions of 31% and 69%, respectively. This indicates that these molecular segments predominantly populate the vacant electronic states, accentuating their potential as electron acceptors during molecular interchanges. Notably, the phenyl fragment (A ring, reference Section 2.4.5.) offers minimal contributions to both the HOMO and LUMO, as visualized in Figure 15.

#### 2.4.5. Evaluating the Aromaticity and π–π Stacking Capability of the Analyzed Molecule

The objective of this study was to quantitatively evaluate the aromatic nature of the molecule under consideration. To achieve this, various aromaticity indices, both electronic and geometric-based, were used. The measurements obtained from these indices are displayed in Table 13. Figure 17 presents the molecular structure diagrams, with distinct labeling for rings and pseudorings. The results reveal that the Harmonic Oscillator Model of Aromaticity (HOMA) values for the three rings in the molecule—the phenyl ring (A), the diphenylnicotinonitrile six-membered ring (B), and the second phenyl ring (C)—are 0.995029, 0.961494, and 0.993422, respectively. The HOMA values for the phenyl rings A and C are particularly noteworthy as they are significantly close to 1, suggesting a robust aromatic character for these two rings. An essential aspect of the aromaticity analysis is the Bird index calculation for the benzene rings (A and C), which are 97.17 and 96.64, respectively. These values are markedly closer to the ideal value of 100 compared to the Bird index for the pyridine ring (B), which is only 87.62. The difference in aromaticity among the rings can be attributed to the disturbance in the cyclic π-electron delocalization. This disturbance is caused by the effect of a phenyl group without substitution, which seems to have a more significant impact on the pyridine ring.

The Shannon aromaticity indices (SAs) for rings A and C, as outlined in Table 13, support the aromatic nature of these rings, as they are less than the defined threshold (SA < 0.003). The antiaromatic behavior of the central pyridine ring, as indicated by its lower HOMA value, is further reinforced by the electronic Shannon index. Its corresponding SA value for ring B (0.002116365) is less than the antiaromatic benchmark of 0.005. The curvature of the electron density measurements offers another layer of validation for the aromaticity assessments. A more negative curvature correlates with increased aromaticity, consistent with the other criteria of aromaticity.

Lastly, the Long-Range Order Parameter (LOLIPOP) is particularly enlightening, given the direct correlation established between its values and π-stacking ability. The inverse relationship between LOLIPOP values and π-depletion propensity implies that a lower LOLIPOP value suggests a stronger π-stacking capacity. Therefore, ring B, with its lower LOLIPOP value compared to rings A and C, possesses a stronger propensity for π-stacking, potentially making it more interactive with other aromatic systems and more suitable for applications requiring π interactions [41,42].

#### 2.4.6. Electrostatic Potential Representation of the Molecule (MEP)

**Figure 18:**
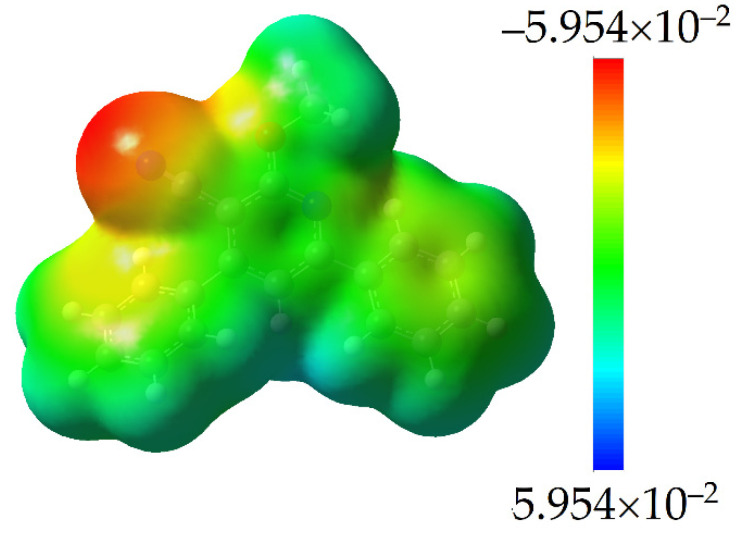
Molecular electrostatic potential map with color gradient for the molecule, ranging from −7.796 × 10^−2^ au to +6.796 × 10^−2^ au.

In the analysis of the molecular electrostatic potential (MEP) for 2-methoxy-4,6-diphenylnicotinonitrile, the charge distribution across the molecule offers insights into specific reactivity sites, as visualized in Figure 18. The MEP values range between −5.954 × 10^−2^ and +5.954 × 10^−2^ atomic units (au). Intensely red regions represent areas with pronounced negative MEP values, signifying electron-rich domains. Specifically, these nucleophilic sites are evidenced in areas surrounding the nitrogen atom of the cyano group, rendering it susceptible to electrophilic attacks. On the other hand, deep blue regions indicate areas with high positive MEP values, marking electron-deficient or electrophilic sites. Several hydrogen atoms within the molecule lie in these blue domains, suggesting that they are potential targets for interactions with electron-rich nucleophiles. Intermediate green regions on the MEP map demarcate zones of relative electronic stability, signifying areas with balanced charge distributions and, consequently, reduced reactivity.

Comparing this MEP analysis with the Frontier molecular orbitals (FMOs) offers an in-depth view of the molecule’s reactivity. While the FMOs provide a picture of the molecule’s possible electronic transitions, especially transitions between the HOMO and LUMO, the MEP directly visualizes areas prone to specific chemical interactions. For instance, regions identified as nucleophilic in the MEP analysis might correlate with areas where the HOMO is densely populated, while electrophilic regions might align with areas dominated by the LUMO. Together, the MEP and FMO analyses can elucidate potential bond interactions, further refining the understanding of the molecule’s interactions in different chemical environments [29,43,44].

### 2.5. Molecular Docking Studies

Molecular docking simulations offer insights into the binding characteristics and interactions between molecules and their target proteins, shedding light on the structure–activity relationships. In this research, docking studies employed the MOE 2015.10 software, sourced from the Chemical Computing Group Inc., Montreal, QC, Canada. A validation procedure was executed by re-docking the target molecule, lipoprotein-associated phospholipase A2 (Lp-PLA2) and its co-crystalline ligand to ensure accuracy in predicting binding orientations.

**Figure 19:**
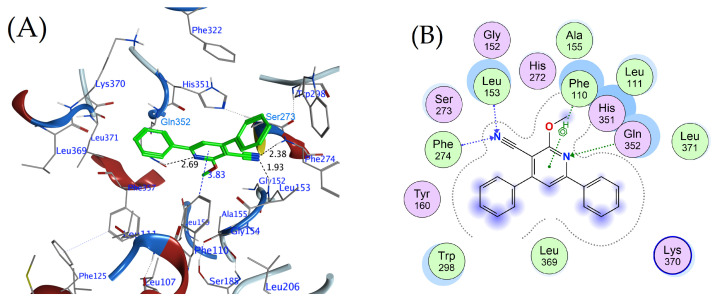
Binding interactions of the title compound with Lp-PLA2 (PDB ID: 6M07): (**A**) 3D representation and (**B**) 2D schematic.

The title compound was subjected to molecular docking against Lp-PLA2. The rationale behind this selection of the target stemmed from research by Jackson et al. [11], which highlighted the activity of molecules belonging to the same class as our title compound. The results showed noteworthy interactions between the title compound and several amino acids in the active site of Lp-PLA2. For instance, the nitrogen atom (N19) of the pyridine group in the title compound established a hydrogen bond interaction with GLN 352’s NE2 atom, while the nitrogen (N36) of the cyano group showed interactions with both LEU 153 and PHE 274. This interaction agrees with the conclusions of the MEP and FMO studies. Furthermore, the 6-ring (pyridine ring) of the title compound demonstrated a π–H interaction with PHE 110 (Figure 19A,B and Table 14). This interaction agrees with the conclusions of the aromaticity study. These specific interactions play a crucial role in determining the molecule’s binding affinity and stability.

Delving deeper into the title compound’s molecular properties, the electrostatic potential representation (MEP) offers an understanding of the molecule’s electrostatic attributes, revealing regions of positive and negative electron density. This can elucidate how the molecule might interact with a target protein’s polar or charged residues. Concurrently, examining the frontier molecular orbitals (FMOs) can shed light on the compound’s reactive regions and potential electron donor or acceptor sites, which can further contribute to its binding mode and strength of interaction with Lp-PLA2.

### 2.6. Molecular Dynamic Simulation

#### 2.6.1. Root Mean Square Deviation (RMSD)

Analysis of the molecular dynamic simulation’s root mean square deviation (RMSD) provides insights into the structural flexibility and conformational changes of the title molecule when in complex with a protein, as compared to the native protein’s inherent flexibility.

**Figure 20:**
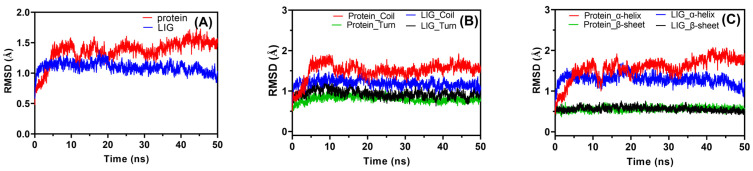
RMSD analysis of the title molecule’s interaction with the protein. (**A**) presents overall protein stabilization upon ligand binding. (**B**) emphasizes enhanced stabilization in coil and turn regions of the complex, while (**C**) depicts consistent beta-sheet flexibility and notable alpha-helix stabilization.

For the protein complexed with the title molecule, the average RMSD value was 1.098, suggesting a stable interaction with the protein. This is further supported by the relatively small standard deviation (0.08). Interestingly, this value is slightly lower than that observed for the native protein, which had a mean RMSD of 1.366, indicating that the protein structure becomes somewhat more stabilized upon interaction with the title molecule (Figure 20A).

Delving deeper into the secondary structures, the alpha-helix in the complex showed a mean RMSD of 1.292, which is significantly lower than the 1.57 observed for the native protein, suggesting that the helical regions might experience more pronounced stabilization due to the ligand binding. Conversely, the beta-sheet regions seem to maintain similar flexibility in both the complex (RMSD of 0.559) and the native protein (RMSD of 0.56) (Figure 20C).

For the turn regions of the complex, the RMSD mean was 0.934, higher than the 0.805 seen in the native protein. This suggests that the ligand binding might be introducing slight perturbations or flexibility in the turn regions. The coil regions in the complex presented an RMSD of 1.193, which, like the alpha-helix, is lower than the native protein’s RMSD of 1.505 (Figure 20B). This highlights a trend where specific structural domains, like the alpha-helix and coil, become more stabilized upon ligand binding, which could be crucial in understanding the molecular underpinnings of ligand recognition and binding specificity.

#### 2.6.2. Root Mean Square Fluctuations (RMSF)

Root mean square fluctuation (RMSF) values offer a glimpse into the flexibility exhibited by individual amino acids in a given protein. Typically, the amino acid residues interacting directly with the ligand tend to have diminished RMSF values. The rationale behind this is straightforward: the ligand’s interaction enforces a certain level of rigidity on the protein, and, consequently, residues exhibiting low RMSF values can indicate a strong restrictive influence by the ligand. This, in turn, might suggest that the ligand has higher inhibitory potential against its protein target [45].

**Figure 21:**
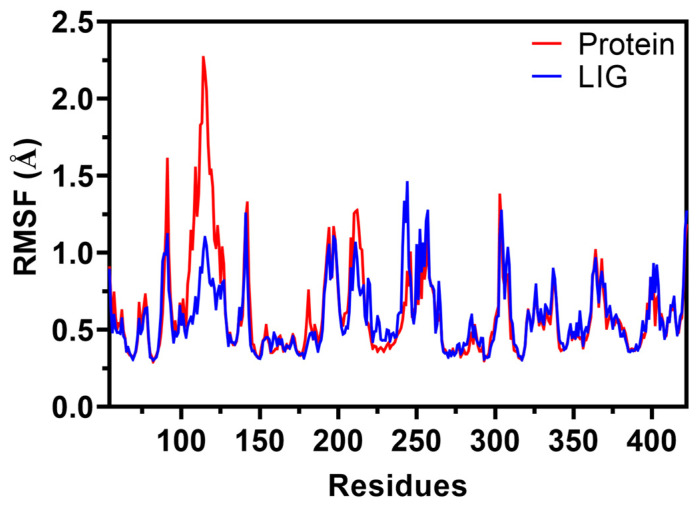
RMSF plot for a 50 ns MDS of both native and complex proteins. The *x*-axis denotes the protein residues while the *y*-axis showcases the RMSF values (in nm). Colors are differentiated as follows: native (in red) and complex (in blue).

In our current investigation, as depicted in Figure 21, the molecular dynamic simulation (MDS) analysis of Lp-PLA2 interacting with the title compound illustrates notably subdued fluctuations among individual amino acids, especially when juxtaposed against other simulation data. Remarkably, amino acids in the region of 106–120 showed the most pronounced reduction in fluctuations compared to their counterparts in native protein simulations. For context, the native protein Lp-PLA2 exhibited an RMSF value of 0.293 Å for Arg-82, a value of 1.243 Å for Phe-110, and the peak value being 2.27 Å for His-114. In stark contrast, when bound to the title compound, these values shift to a mere 0.303 Å for Arg-82, 0.61 Å for Phe-110, and 1.01 Å for His-114.

These observations not only underscore the ligand’s ability to impart rigidity to the interacting residues but also suggest that these residues, which engage through pi–H interactions with Phe-110 (Figure 19), resonate well with findings from the Hirshfeld surface analysis and density functional theory. Collectively, based on the presented RMSF data, the title compound emerges as a potent contender, manifesting remarkable restraints on the target protein’s flexibility and implying its high inhibitory capability against Lp-PLA2.

#### 2.6.3. Radius of Gyration (Rg)

The radius of gyration (Rg) is a pivotal parameter in molecular dynamic simulations that provides insights into the overall compactness and shape of a protein. Essentially, it evaluates the mass-weighted root mean square distance of a collection of atoms (in this context, the protein’s backbone) from their collective center of mass. A consistent Rg value throughout a simulation suggests structural stability, while significant deviations might indicate conformational changes or unfolding events.

**Figure 22:**
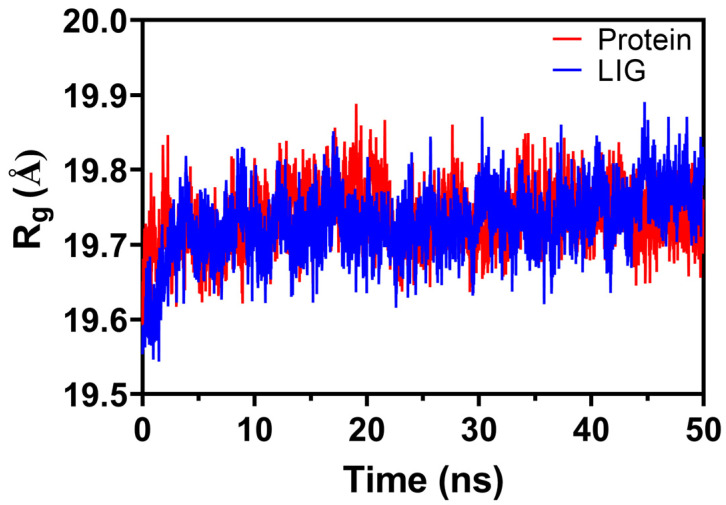
Plot representing the radius of gyration over a 50 ns MD simulation duration. The red curve denotes the native protein, while the blue curve showcases the ligand-bound complex.

In our analysis, the Rg values for both the native protein and the complex system were found to be remarkably close, with the native protein registering an Rg value of 19.737 nm and the complex system slightly less at 19.728 nm (Figure 22). Such proximate values suggest that the binding of the ligand (forming the complex) did not lead to any substantial alteration in the overall compactness of the protein, implying that the inherent structural integrity of the protein was retained after ligand binding. This observation is of particular importance, as it indicates that the ligand’s interaction with the protein did not induce any drastic structural perturbations that might have otherwise compromised the protein’s functionality or stability.

#### 2.6.4. Hydrogen Bond Analysis

**Figure 23:**
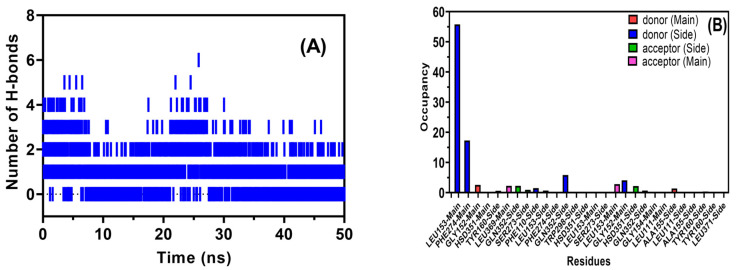
Depiction of hydrogen bonds between the protein and ligand throughout the MD simulation. Panel (**A**) illustrates the number of hydrogen bonds, while Panel (**B**) showcases the hydrogen bond occupancy (%) and the involved residues.

Hydrogen bonds play a pivotal role in stabilizing protein–ligand interactions. In the 50 ns MDS study, there were instances of up to six hydrogen bonds, but, on average, one bond persisted throughout (Figure 23A). The consistent hydrogen bond suggests a stable interaction over the simulation duration. In Figure 23B, detailed analysis reveals that LEU153, contributing mainly through the main chain, is the most dominant in forming hydrogen bonds, showcasing an occupancy of 55.78%. Other residues, such as PHE274-Main and GLY152-Main, exhibit transient interactions. Both main and side chain residues participated in these bonds, underscoring the complexity of the ligand–protein interaction dynamics in the system.

### 2.7. ADMET and Physicochemical Property Prediction

#### 2.7.1. Physicochemical Properties of 2-Methoxy-4,6-Diphenylnicotinonitrile

The compound 2-methoxy-4,6-diphenylnicotinonitrile exhibits a molecular weight of 286.11 Da, fitting comfortably within the acceptable range for drug-like molecules. It possesses three hydrogen bond acceptors and no donors, aligning with typical drug profiles. Its moderate flexibility is demonstrated by its three rotatable bonds, and its structural integrity is further suggested by its 3 rings and 19 rigid bonds. The compound’s topological polar surface area (TPSA) of 45.91 Å^2^ suggests a good balance between membrane permeability and solubility. However, concerns arise from its aqueous solubility value (logS) of −5.979 and high n-octanol/water distribution coefficient (logP) of 4.693, indicating limited water solubility and increased lipophilicity, respectively.

In the context of drug development rules, the compound adheres to Lipinski’s Rule of Five, indicating that it possesses characteristics commonly associated with orally active drugs. However, it does not conform to the Pfizer and GSK rules, suggesting potential challenges in drug development according to these specific guidelines.

In essence, while 2-methoxy-4,6-diphenylnicotinonitrile showcases properties indicative of drug-like molecules, its pronounced lipophilicity and reduced solubility might pose challenges in drug formulation.

#### 2.7.2. ADMET Prediction of 2-Methoxy-4,6-Diphenylnicotinonitrile

The title compound’s absorption properties present a mixed profile. Its Caco-2 permeability of −4.641 suggests excellent intestinal absorption potential. Similarly, the MDCK permeability value of 3.12 × 10^−5^ falls within the excellent range, indicating favorable membrane permeability. However, there are concerns regarding its interaction with P-glycoprotein, as it is indicated to be a poor inhibitor with a score of 0.92, although it is not likely to be a substrate. While the human intestinal absorption (HIA) rating of 0.004 indicates excellent absorption potential, the compound’s predicted human oral bioavailability (F20%) is considered poor, with a score of 0.774. Conversely, its bioavailability (F30%) score of 0.008 categorizes it as excellent. These results suggest that while the compound has promising absorption properties in certain domains, its potential as an orally bioavailable drug might be restricted due to its P-glycoprotein interactions and inconsistent bioavailability predictions.

The title compound exhibits plasma protein binding (PPB) of 100.20%, exceeding the preferred limit, which might impede its effectiveness. With a volume of distribution (VD) of 0.681, it falls within the excellent range, suggesting primary confinement within the vascular compartment. The compound’s potential to penetrate the blood–brain barrier (BBB) is moderate, with a value of 0.38, indicating possible accessibility to the central nervous system. However, its low fraction unbound in plasma (Fu) at 1.10% suggests limited bioactive availability, potentially affecting its pharmacological action.

The title compound’s interactions with cytochrome P450 (CYP) enzymes suggest potential metabolic pathways and drug–drug interactions. It shows strong potential to inhibit CYP1A2 (0.96), CYP2C19 (0.907), and CYP2C9 (0.916), indicating possible interference with drugs metabolized by these enzymes. Additionally, the compound is a probable substrate for CYP1A2 (0.431), CYP2C9 (0.885), and CYP3A4 (0.251). However, it has a minimal inhibitory effect on CYP2D6 (0.001) and CYP3A4 (0.136), reducing the potential interactions with drugs processed by these enzymes. This metabolic profile suggests the need for the title compound’s careful co-administration with other medications due to potential interactions.

The title compound’s excretion properties give insights into its pharmacokinetics and potential therapeutic utility. The drug clearance (CL) value is 8.261, which is beyond the excellent threshold (≥5), indicating that the compound is efficiently cleared from the system. Meanwhile, its half-life (T1/2) stands at 0.081, falling within the excellent range (0–0.3). This suggests that the drug is rapidly metabolized and has a short duration of action in the body. Such a quick turnover might necessitate frequent dosing to maintain therapeutic levels, depending on its intended application.

Evaluating the toxicity properties of the title compound provides essential insights into its safety profile. The predicted maximum recommended daily dose (FDAMDD) is 0.901, which is classified as poor based on its value falling in the 0.7–1.0 range. This suggests caution when considering dosage recommendations. While it shows excellent potential as a non-skin sensitizer, with a value of 0.058, its carcinogenicity potential is medium at 0.55, indicating a moderate risk associated with prolonged exposure. The compound is very promising in terms of non-eye corrosion, having an excellent value of 0.006. However, it presents a high risk for eye irritation, as evidenced by its poor score of 0.961, suggesting the necessity for precaution when handling or administering it. Additionally, the respiratory toxicity value of 0.472 falls within the medium range, indicating potential concerns for respiratory exposure. Taken together, while the title compound has certain favorable toxicity profiles, there are notable areas of concern that warrant thorough evaluation before therapeutic application.

## 3. Materials and Methods

### 3.1. Synthesis

**Scheme 1:**
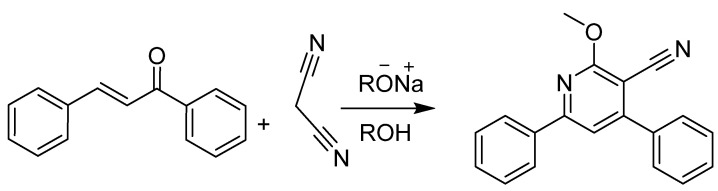
Synthesis of 2-methoxy-4,6-diphenylnicotinonitrile.

The synthesis of the title compound was meticulously carried out as delineated in Scheme 1, referencing the established protocol detailed in the scientific literature by Al-Arab [46]. Following synthesis, the compound was subjected to a crystallization process using acetone as the solvent. This resulted in the formation of slender, colorless crystalline plates [24].

### 3.2. Single-Crystal X-ray Diffraction

The molecular structure of the designated compound was unequivocally elucidated through single-crystal X-ray diffraction analysis. Comprehensive details of this structural determination are documented in the work conducted by Mague et al. [24].

The structural solution was achieved via direct methods with SHELXS-97 [47], while geometrical parameters and weak interactions were analyzed using Olex2-1.5-alpha [48]. Visualization was facilitated through software suites such as MERCURY 3.1 [49] and DIAMOND 4.5 [50], with atomic-level details derived from ORTEP3 [51]. Pertinent structural details are catalogued in Table 1, and specific bond distances and angles can be found in Section 2.4.1.

## 4. Computational Details

### 4.1. Hirshfeld Surfaces

The in-depth analysis of 2-methoxy-4,6-diphenylnicotinonitrile hinged on the utilization of the Hirshfeld surface methodology, which brought forth an understanding of several molecular attributes. Aspects like dnorm, di, de, curvedness, and the shape index were thoroughly mapped using the CrystalExplorer 21.5 software [20,29,30,52]. Specifically, dnorm presented insights into normalized contact distances between atomic pairs in the crystal structure [53,54], whereas the di and de surfaces pinpointed the closest interior and exterior elements to the Hirshfeld molecular surface [55]. The color designations represent the relative distances between X and Y: red indicates that the sum of their van der Waals radii is less than the established separation (dnorm < 0), blue signifies that it is greater (dnorm > 0), and white means that it is exactly equal (dnorm = 0) [54].

The equation for dnorm is

(1) $$d_{norm}=\frac{\left(d_{i}-r_{i}^{vdw}\right)}{r_{i}^{vdw}}+\frac{\left(d_{e}-r_{e}^{vdw}\right)}{r_{e}^{vdw}}$$

Here, $r_{i}^{vdw}$, $r_{e}^{vdw}$ represent the van der Waals radii for elements X and Y, respectively, while di and de indicate their corresponding distances.

Curvedness and the shape index [56] were instrumental in capturing the surface curvature and nuanced alterations in surface geometry, respectively [38,57]. The innovative two-dimensional fingerprint plots enabled a detailed depiction of inter-atomic contact contributions towards the Hirshfeld surface [58].

The analysis was executed using the CrystalExplorer 21.5 software [59], integrated with the TONTO system [59,60]. For dnorm surfaces, a standardized color gradient was employed, spanning from −0.15 atomic units (a.u.), represented in red, to 1.15 a.u., showcased in blue. Concurrently, the mapping parameters for shape index and curvedness were set to range from −1.0 to 1.0 and −4.0 to 0.4 Å, respectively.

Recent advancements have enabled the detailed examination of intermolecular interactions within crystals using the Hirshfeld surface analysis. One significant outcome is the ability to map the molecular electrostatic potential on these surfaces, giving a direct view of specific intermolecular interactions, particularly those of the form D-H⋯A [24]. Gavezzotti’s PIXEL method has garnered attention for its capability to provide dependable intermolecular energies using a combination of non-empirical and semi-empirical methods [61]. While other quantum mechanical techniques have been utilized, such as those by Shishkin et al., they often require more computational time, limiting their application to extensive molecular crystal studies [62].

Our research aimed to fine-tune an effective model to estimate intermolecular interaction energies. A prevalent model for such calculations in both organic and inorganic molecular crystals is CrystalExplorer. This approach adopts Gavezzotti’s interaction energy formula:

(2) $$E_{tot}=E_{ele}+E_{pol}+E_{dis}+E_{rep}$$

In our methodology, we calculated the interaction energies between different molecules in reference to the primary molecule within the crystal structure. The total interaction energy is computed from several components: classical electrostatic interactions [33,63], polarization energies based on recommended isotropic atomic polarizabilities, dispersion corrections incorporating intermolecular atomic pairs, and repulsion energies calculated from charge distributions. Utilizing the CrystalExplorer21.5 software, we engaged its accurate energy model, particularly suitable for our title compound, which consists of an asymmetric unit housing two molecules. Computations for molecules A and B were executed separately, employing the crystallographic information file (.cif) as input. Each calculation involved the primary molecule being surrounded by a cluster of fifteen other molecules, totaling 465 atoms, within a 3.8 Å radius [33,64].

### 4.2. Density Functional Theory (DFT) Calculations

In the current study, detailed computational analyses of the title compound were conducted as follows. Density functional theory (DFT) calculations were initiated using the crystallographic information files (CIF) from the single-crystal X-ray data previously reported (ccdc code 983247) by Mague et al. [24]. These provided the input geometries. Geometry optimization of different isolated monomer models was undertaken in the gaseous phase utilizing the Gaussian 09, Rev D.01 software package [65]. For visualization, data analysis, modification, and the exportation of results, the GaussView 6.0 program [66] was employed.

The computational approach integrated the wB97XD functional from Head-Gordon and associates, which incorporates Grimme’s D2 dispersion model and long-range corrections [23]. Additionally, the renowned B3LYP functional [67] was used for geometric structure optimization. Among these, wB97X-D was deemed superior in capturing hydrogen bonding interactions [29,52,68,69].

Frequency calculations on the optimized geometries confirmed all stationary points as genuine minima, evidenced by zero imaginary frequencies. These validated geometries were then subjected to single-point (SP) calculations at the identical theoretical level to deduce reactivity descriptors, frontier molecular orbitals (FMOs), the molecule’s electrostatic potential (MEP), and aromaticity attributes [35,36,70].

To compute both global and localized chemical reactivity descriptors, the concepts of conceptual DFT (CDFT), also referred to as chemical reactivity theory (CRT), were employed through the MULTIWFN program [71,72,73,74]. The evaluation of electrophilic $P_{k}^{+}$ and nucleophilic $P_{k}^{-}$ Parr functions was achieved by analyzing the Mulliken atomic spin density (ASD) of both the radical anion and cation, all of which were derived from optimized neutral geometries [71]. For SP energy calculations, an unrestricted open-shell DFT approach was implemented, specifically WB97XD/6-311G(d,p). This utilized combinations (charge and multiplicity) designated as (+1, 2) for cations and (−1, 2) for anions.

Aromaticity indices and LOLIPOP metrics for the title compounds were calculated via the MULTIWFN program [71]. Finally, time-dependent density functional theory (TD-DFT) calculations were performed using the Gaussian software to determine electronic excitation energies and the associated molecular orbital transitions. The resulting output files were subsequently analyzed with the GaussSum 3.0 software. This analysis allowed for the extraction of molecular orbital contributions associated with specific functional groups within the molecule, as elaborated in references [75,76]. Moreover, GaussSum 3.0 was utilized to compute the density of states (DOS), the partial density of states (PDOS) spectra, and UVData. These data are presented in Figure 15 and Table 11. It is imperative to acknowledge that while TD-DFT provides a credible perspective on excitation energies, emerging range-separated DFT techniques are expected to offer superior accuracy in their calculations in subsequent revisions [77,78].

## 5. In Silico Methods

### 5.1. Molecular Docking

The Protein Data Bank (PDB) was used to obtain the crystal structure of lipoprotein-associated phospholipase A2 (Lp-PLA2), which was given the ID 6M07 [79]. In this study, the binding of the ligand title molecule to the receptor was examined using the molecular docking software MOE 2015. First, the shape of the ligand was improved as much as possible using the MMFF94x force field. Using the MOE 2015 model, the protonation states of the structure were set and partial charges were given. Next, water molecules were taken out of the PDB file for the protein. The Amber10 forcefield was used to fix any missing side chains and residues and reduce the energy of the proteins. The groups that were made were saved in the MDB format. Then, the best cluster was chosen based on the scoring energy, how the ligand interacted with key residues, and how the ligand was oriented.

### 5.2. Molecular Dynamic Simulation

In this study, we employed molecular dynamic (MD) simulations to probe the behavior and stability of our top-performing compound when bound to the lipoprotein-associated phospholipase A2 (Lp-PLA2) protein (PDB ID: 6M07) [79]. Our selection was based on both the binding scores and modes observed during docking. We adopted methodologies from our prior research [30,80,81]. These simulations were executed using the NAMD program [82]. To initiate the simulation, we acquired the requisite configuration files from the CHARMM-GUI website [83,84]. We further refined the properties of our ligands with the CHARMM general force field (CGenFF) tool accessible online. The entire protein–ligand system was immersed in a water environment using the TIP3P model. The system then underwent an energy minimization process spanning 90 million steps to ensure stability, which continued for a 50 ns timescale using the NVT ensemble. To comprehend and interpret the results, we used the VMD viewer [85], which provided us with visual and quantitative insights into the simulation. The structural deviations and flexibility during the simulations were quantified by calculating root mean square deviation (RMSD) and root mean square fluctuation (RMSF) values. Furthermore, we determined the molecule’s compactness using parameters like Rg and SASA. Hydrogen bond analysis was performed to gain deeper insights into the binding behavior and conformational alterations of the protein–ligand complex.

### 5.3. Drug-Likeness and ADMET Prediction

Utilizing the ADMETLab2.0 online tool available at https://admetmesh.scbdd.com/service/screening/index (accessed on 6 September 2023), we assessed the drug-likeness and ADMET profiles of the designated compound. After inputting the compound’s molecular structural data in either 2D or 3D format, the tool evaluated its drug-likeness based on established criteria, notably Lipinski’s Rule of Five. The platform’s ADMET prediction module, which integrates in-silico models with machine learning algorithms, subsequently provided insights into the compound’s absorption, distribution, metabolism, excretion, and toxicity properties, furnishing a comprehensive understanding of its potential therapeutic viability.

## 6. Conclusions

The comprehensive study of 2-methoxy-4,6-diphenylnicotinonitrile reveals a unique molecular arrangement, emphasizing the compound’s structure, governed by a pyridine ring flanked by two phenyl rings. This configuration presents a fascinating interplay of forces, with weak intermolecular interactions like H⋯H contacts and substantial π–π stacking due to p-orbital overlaps in adjacent aromatic rings. While these interactions might be inherently weak, they serve as a cornerstone for the structural stability and crystalline properties. Energy framework insights underscore the prominence of dispersion intermolecular interactions, solidifying the crystal structure’s foundation. DFT analyses further accentuate the aptness of the WB97XD function over B3LYP in capturing the molecule’s intricate geometric nuances. Alongside this, reactivity descriptors illuminate the molecule’s inherent chemical behavior, highlighting the principal reactive sites. Moreover, frontier molecular orbitals (HOMO and LUMO) emerge as pivotal in determining the electronic demeanor and robustness of the compound, with a noteworthy HOMO–LUMO gap reflecting its stability. Aromaticity investigations corroborate the dominant aromatic characteristics of the phenyl rings, whereas the pyridine ring displays subdued aromaticity, attributed to disruptions in cyclic π-electron delocalization. The molecule’s π-stacking capabilities are prominently exhibited by the pyridine ring, demonstrating its proclivity for π–π interactions. Lastly, the MEP analysis meticulously maps out the molecule’s electrophilic and nucleophilic sites, providing an intricate framework of its interaction dynamics in varied chemical contexts.

Molecular docking studies reveal significant interactions between the title compound and Lp-PLA2, specifically noting hydrogen bonding and π–H interactions. Molecular dynamic simulations, including RMSD and RMSF analyses, suggest that the title compound has a stabilizing effect on certain secondary structures of Lp-PLA2. The title compound does not drastically change the overall compactness of Lp-PLA2 upon binding, as evidenced by the radius of gyration. Hydrogen bond analysis underpins the molecule’s stable interaction over the simulation period. The compound 2-methoxy-4,6-diphenylnicotinonitrile showcases properties indicative of drug-like molecules. However, its pronounced lipophilicity and reduced solubility might pose challenges in drug formulation. ADMET prediction portrays a mixed profile, indicating potential challenges in oral bioavailability due to P-glycoprotein interactions. Additionally, the compound’s interactions with specific cytochrome P450 enzymes suggest potential metabolic pathways and drug–drug interactions. In essence, while the compound offers promising interactions and effects on Lp-PLA2, its pharmacological applications require further consideration.

## Figures and Tables

**Table 1:** Crystal structure and refinement details for 2-methoxy-4,6-diphenylnicotinonitrile.

Parameter	Data
Identification code	983247
Empirical formula	C_19_H_14_N_2_O
Formula weight	286.32
Temperature/K	100(2)
Crystal system	orthorhombic
Space group	P2_1_2_1_2
a/Å	15.0686(16)
b/Å	24.327(3)
c/Å	3.8986(4)
α/°	90.00
β/°	90.00
γ/°	90.00
Volume/Å^3^	1429.1(3)
Z	4
ρ_calc_g/cm^3^	1.331
μ/mm^−1^	0.084
F(000)	600.0
Crystal size/mm^3^	0.22 × 0.11 × 0.06
Radiation	MoKα (λ = 0.71073)
2Θ range for data collection/°	3.18 to 56.92
Index ranges	−19 ≤ h ≤ 20, −31 ≤ k ≤ 31, −5 ≤ l ≤ 5
Reflections collected	12356
Independent reflections	3344 [R_int_ = 0.0465, R_sigma_ = 0.0411]
Data/restraints/parameters	3344/0/200
Goodness-of-fit on F^2^	1.036
Final R indexes [I ≥ 2σ (I)]	R_1_ = 0.0404, wR_2_ = 0.0880
Final R indexes [all data]	R_1_ = 0.0481, wR_2_ = 0.0933
Largest diff. peak/hole/e Å^−3^	0.20/−0.21

**Table 2:** Hydrogen bonding in cocrystal 2-methoxy-4,6-diphenylnicotinonitrile.

Number		Angle/°	Length/Å	Length-VdW	Symm. op. 1	Symm. op. 2
1	H6B⋯H10	138.99	2.364	−0.036	x,y,z	1 − x,−y,−1 + z
2	H6A⋯H6A	149.55	2.372	−0.028	x,y,z	1 − x,−y,z
3	H17⋯C10	159.57	2.824	−0.076	x,y,z	1.5 − x,1/2 + y,−z
4	N2⋯H19	136.08	2.749	−0.001	x,y,z	−1/2 + x,1/2 − y,−1 − z
5	N2⋯H2	176.33	2.702	−0.048	x,y,z	−1/2 + x,1/2 − y,−z
6	N2⋯H13	159.25	2.663	−0.087	x,y,z	−1/2 + x,1/2 − y,−z
7	H12⋯H16	126	2.398	−0.002	x,y,z	1/2 + x,1/2 − y,1 − z

**Table 3:** Statistical analysis of Hirshfeld surface properties for the molecular system.

Name	Mean+	Mean−	Pi	Sigma+	Sigma−	SigmaT	Nu
di	1.67	~	0.216	5.08 × 10^9^	~	2.16 × 10^14^	0
de	1.69	~	0.207	5.16 × 10^9^	~	2.20 × 10^14^	0
dnorm	0.45	−0.0287	0.2	3.63 × 10^8^	45.3	1.54 × 10^13^	6.92 × 10^−10^
Shape Index	0.515	−0.407	0.408	2.39 × 10^8^	2.61 × 10^7^	7.49 × 10^12^	0.0419
Curvedness	0.0626	−1.04	0.438	152	1.93 × 10^9^	8.19 × 10^14^	3.68 × 10^−10^

**Table 4:** Fragment patch information for 16 fragment patches of Figure 7.

Color of the Area	Area Number.	Area/Å^2^	Color of the Area	Area Number	Area/Å^2^
	1	7.7		9	12.4
	2	69.9		10	30.8
	3	22.2		11	6.0
	4	31.7		12	9.1
	5	7.7		13	9.4
	6	10.6		14	11.2
	7	10.6		15	71.7
	8	9.6		16	8.7

**Table 5:** Percentage of total surface area representing close interactions between internal and external atoms.

Inside Atom	Outside Atom				
	O	N	C	H	
C	0.1	1.5	15.5	7.7	24.9
H	2.7	6.5	5.1	48.5	62.8
N	0.1	0	1.3	7.6	9
O	0	0.1	0.1	3.1	3.3
	2.9	8.2	22.1	66.9	

**Table 6:** The component is presented in the form of interaction energies for the tested molecule, measured in kilojoules per mole (kJ/mol). ‘N’ represents the number of surrounding molecules relative to the original molecule, identified by the respective color code. ‘R’ signifies the distance between the centroids of the molecules, calculated as the mean atomic position.

	N	Symop	R	Electron Density	E_ele_	E_pol_	E_dis_	Er_ep_	E_tot_
	2	−x, −y, z	10.44	B3LYP/6-31G(d,p)	−0.9947	−0.4697	−7.6196	5.1944	−4.8259
	2	x, y, z	3.90	B3LYP/6-31G(d,p)	−2.0088	−3.5951	−95.9593	55.0986	−54.3109
	1	−x, −y, z	9.69	B3LYP/6-31G(d,p)	−2.7551	−0.8832	−19.1408	10.9829	−13.4501
	2	−x + 1/2, y + 1/2, −z	12.77	B3LYP/6-31G(d,p)	−0.8316	−0.3984	−8.7222	0.0000	−8.7693
	2	−x + 1/2, y + 1/2, −z	13.03	B3LYP/6-31G(d,p)	−2.8150	−0.2619	−7.7392	0.0000	−9.9093
	2	x + 1/2, −y + 1/2, −z	8.28	B3LYP/6-31G(d,p)	−13.6349	−6.3419	−28.7381	26.3618	−27.8500
	2	x + 1/2, −y + 1/2, −z	9.60	B3LYP/6-31G(d,p)	−7.7203	−3.2340	−8.5227	5.6320	−14.4981
	2	x + 1/2, −y + 1/2, −z	8.67	B3LYP/6-31G(d,p)	0.1625	−0.6978	−11.9824	6.7784	−6.5917
	1	−x, −y, z	14.38	B3LYP/6-31G(d,p)	1.5232	−0.1605	−3.1131	0.0000	−1.2192

**Table 7:** Comparison of bond lengths (in Ångstroms) between experimental values from X-ray diffraction and theoretical predictions using B3LYP, WB97XD, and B3PW91 with the 6-311G(d,p) basis set for the compounds.

Length/Å	Experimental	Theoretical (DFT)			Absolute Error		
Atom	SC-XRD	B3PW91	wb97xd	b3lyp	B3PW91	wb97xd	b3lyp
O1―C5	1.3583(19)	1.331	1.3397	1.43	0.0273	0.0186	0.0717
O1―C6	1.437(2)	1.4243	1.4345	1.43	0.0127	0.0025	0.007
N1―C1	1.359(2)	1.3419	1.3485	1.3948	0.0171	0.0105	0.0358
N1―C5	1.311(2)	1.3139	1.317	1.3951	0.0029	0.006	0.0841
N2―C7	1.147(2)	1.1516	1.1558	1.1466	0.0046	0.0088	0.0004
C1―C2	1.388(2)	1.3888	1.395	1.3952	0.0008	0.007	0.0072
C1―C8	1.485(2)	1.4846	1.4843	1.54	0.0004	0.0007	0.055
C2―C3	1.397(2)	1.3956	1.4	1.3947	0.0014	0.003	0.0023
C3―C4	1.400(2)	1.3966	1.4057	1.3954	0.0034	0.0057	0.0046
C3―C14	1.487(2)	1.4829	1.4851	1.54	0.0041	0.0019	0.053
C4―C5	1.411(2)	1.4124	1.4198	1.3948	0.0014	0.0088	0.0162
C4―C7	1.435(2)	1.4272	1.4243	1.54	0.0078	0.0107	0.105
C8―C9	1.398(2)	1.3964	1.4025	1.3952	0.0016	0.0045	0.0028
C8―C13	1.397(2)	1.3966	1.4028	1.3948	0.0004	0.0058	0.0022
C9―C10	1.385(2)	1.3865	1.3899	1.3947	0.0015	0.0049	0.0097
C10―C11	1.390(2)	1.39	1.3939	1.3954	0	0.0039	0.0054
C11―C12	1.386(2)	1.3894	1.3934	1.3948	0.0034	0.0074	0.0088
C12―C13	1.380(2)	1.3876	1.3907	1.3951	0.0076	0.0107	0.0151
C14―C15	1.399(2)	1.395	1.4009	1.3952	0.004	0.0019	0.0038
C14―C19	1.395(2)	1.396	1.4022	1.3948	0.001	0.0072	0.0002
C15―C16	1.388(2)	1.3878	1.3913	1.3947	0.0002	0.0033	0.0067
C16―C17	1.382(2)	1.3893	1.3929	1.3954	0.0073	0.0109	0.0134
C17―C18	1.386(2)	1.3894	1.3932	1.3948	0.0034	0.0072	0.0088
C18―C19	1.386(2)	1.3875	1.391	1.3951	0.0015	0.005	0.0091
Mean Absolute Error (MAE)					0.004825	0.006538	0.022013

**Table 8:** Comparison of bond angles (in degrees) between experimental values obtained from X-ray diffraction and theoretical predictions using B3LYP, WB97XD, or B3PW91 with the 6-311G(d,p) basis set for the compounds.

Angle/°	Experimental	Theoretical (DFT)			Absolute Error		
Atoms	SC-XRD	B3PW91	wb97xd	b3lyp	B3PW91	wb97xd	b3lyp
C5―O1―C6	116.07(13)	117.6143	118.0351	109.5	1.5443	1.9651	6.57
C5―N1―C1	117.68(14)	118.9425	119.0151	120	1.2625	1.3351	2.32
N1―C1―C2	121.80(15)	121.6597	121.2835	119.9985	0.1403	0.5165	1.8015
N1―C1―C8	115.97(14)	116.191	116.4997	120.0043	0.221	0.5297	4.0343
C2―C1―C8	122.23(14)	122.1361	122.2057	119.9972	0.0939	0.0243	2.2328
C1―C2―C3	120.49(15)	120.1082	120.599	120.0086	0.3818	0.109	0.4814
C2―C3―C4	117.52(15)	117.8936	117.65	119.9942	0.3736	0.13	2.4742
C2―C3―C14	119.74(15)	119.6337	119.6121	120.0128	0.1063	0.1279	0.2728
C4―C3―C14	122.74(15)	122.4719	122.7377	119.993	0.2681	0.0023	2.747
C3―C4―C5	117.62(15)	117.7392	117.5435	119.994	0.1192	0.0765	2.374
C3―C4―C7	123.46(15)	122.9391	123.3874	119.9811	0.5209	0.0726	3.4789
C5―C4―C7	118.89(15)	119.3067	119.0467	120.0249	0.4167	0.1567	1.1349
O1―C5―C4	115.69(14)	116.5456	116.1911	120.0113	0.8556	0.5011	4.3213
N1―C5―O1	119.45(15)	119.8054	119.9158	119.984	0.3554	0.4658	0.534
N1―C5―C4	124.86(15)	123.6463	123.8904	120.0047	1.2137	0.9696	4.8553
N2―C7―C4	178.56(19)	179.8173	179.2402	180	1.2573	0.6802	1.44
C9―C8―C1	120.03(14)	119.5014	119.8124	119.9972	0.5286	0.2176	0.0328
C13―C8―C1	121.52(15)	121.7039	121.764	120.0043	0.1839	0.244	1.5157
C13―C8―C9	118.44(15)	118.7942	118.4223	119.9985	0.3542	0.0177	1.5585
C10―C9―C8	120.84(16)	120.5916	120.7559	120.0086	0.2484	0.0841	0.8314
C9―C10―C11	120.12(16)	120.1896	120.2645	119.9942	0.0696	0.1445	0.1258
C12―C11―C10	119.29(16)	119.6758	119.5661	119.994	0.3858	0.2761	0.704
C13―C12―C11	120.81(17)	120.1707	120.2242	120.0047	0.6393	0.5858	0.8053
C12―C13―C8	120.49(16)	120.5715	120.7626	120	0.0815	0.2726	0.49
C15―C14―C3	121.35(15)	121.4162	121.4486	119.9972	0.0662	0.0986	1.3528
C19―C14―C3	119.47(14)	119.384	119.7933	120.0043	0.086	0.3233	0.5343
C19―C14―C15	119.15(15)	119.193	118.7431	119.9985	0.043	0.4069	0.8485
C16―C15―C14	119.70(16)	120.2317	120.4784	120.0086	0.5317	0.7784	0.3086
C17―C16―C15	120.56(16)	120.2587	120.3076	119.9942	0.3013	0.2524	0.5658
C16―C17―C18	120.19(15)	119.8341	119.7065	119.994	0.3559	0.4835	0.196
C17―C18―C19	119.64(16)	120.0176	120.0971	120.0047	0.3776	0.4571	0.3647
C18―C19―C14	120.72(15)	120.4614	120.6642	120	0.2586	0.0558	0.72
Mean Absolute Error (MAE)					0.42631875	0.386275	1.625831

**Table 9:** Evaluated compounds’ global reactivity descriptor values (expressed in eV), computed utilizing the wB97X-D/6.31G (d,p) theoretical model.

Vertical IP (eV)	Vertical EA (eV)	Mulliken Electronegativity (eV)	Chemical Potential (eV)	Hardness (eV)	Softness (eV^−1^)	Electrophilicity Index (eV)	Nucleophilicity Index (eV)
7.7691	0.3931	4.0811	−4.0811	7.3759	0.1356	1.1290	2.8345

**Table 10:** Computed local electrophilic $P_{k}^{+}$ and nucleophilic $P_{k}^{-}$ Parr functions derived from Mulliken atomic spin densities for the investigated molecules.

Atoms	$P_{k}^{+}$	$P_{k}^{-}$
C1	0.5642	6.829
C2	5.788	−2.458
C3	−3.521	5.293
C4	9.618	5.372
C5	−0.047	−0.941
N7	−0.332	2.558
O8	2.468	−0.084
C9	0.266	0.0119
C13	−2.552	−0.833
N14	4.712	3.193
C15	0.871	−0.194
C16	−0.325	0.841
C17	−0.005	1.176
C18	0.270	−0.366
C20	−0.01592	−0.53473
C22	0.316333	1.631732
C26	3.001526	−0.09676
C27	1.516165	2.361079
C28	1.541635	2.098325
C29	−0.80325	−1.1757
C31	−0.79917	−1.06386
C33	4.678211	3.593401

**Table 11:** Computed absorption wavelengths (λ, in nm), excitation energies (in eV), and oscillator strengths (f) for the subject compound.

No.	Energy (cm^−1^)	Wavelength (nm)	Osc. Strength	Major Contributors	Minor Contributors
1	35,227.88	283.8661	0.5084	HOMO->LUMO (86%)	H–4->LUMO (2%), H–1->LUMO (4%)
2	40,377.73	247.6613	0.0355	H–5->LUMO (38%), H–2->LUMO (19%)	H–4->LUMO (3%), H–4->L+3 (2%), H–3->LUMO (6%), H–2->L+1 (2%), H–1->LUMO (6%), HOMO->L+1 (9%), HOMO->L+3 (4%)
3	40,469.68	247.0986	0.0694	H–5->LUMO (27%), H–1->LUMO (50%)	H–6->LUMO (2%), HOMO->LUMO (4%), HOMO->L+1 (7%)

**Table 12:** Analysis of molecular orbitals, their energy levels, and electron density distribution between C6H5 and C7H3N2O components of the molecule. The table provides detailed data for the highest occupied molecular orbital (HOMO) and the lowest unoccupied molecular orbital (LUMO), alongside other significant orbitals.

	MO	eV	Symmetry	C6H5	C7H3N2O	Accurate Values	
6	L+10	3.74	A	72	28	0.719	0.281
85	L+9	3.59	A	46	54	0.455	0.545
84	L+8	3.32	A	87	13	0.867	0.133
83	L+7	3.12	A	21	79	0.21	0.79
82	L+6	3	A	77	23	0.77	0.23
81	L+5	2.06	A	69	31	0.695	0.305
80	L+4	1.85	A	49	51	0.487	0.513
79	L+3	1.39	A	98	2	0.98	0.02
78	L+2	1.34	A	97	3	0.965	0.035
77	L+1	0.67	A	50	50	0.497	0.503
76	LUMO	−0.4	A	31	69	0.312	0.688
75	HOMO	−8.41	A	34	66	0.342	0.658
74	H–1	−9.04	A	76	24	0.761	0.239
73	H–2	−9.33	A	98	2	0.978	0.022
72	H–3	−9.33	A	99	1	0.986	0.014
71	H–4	−9.6	A	56	44	0.558	0.442
70	H–5	−10.08	A	9	91	0.087	0.913
69	H–6	−10.51	A	29	71	0.287	0.713
68	H–7	−11.37	A	8	92	0.084	0.916
67	H–8	−11.76	A	28	72	0.284	0.716
66	H–9	−11.84	A	74	26	0.74	0.26
65	H–10	−11.93	A	77	23	0.769	0.231

**Table 13:** Computed electronic and geometric aromaticity indices, together with LOLIPOP metric, for the investigated molecules.

Rings	Shannon Aromaticity Index	Electron Density Curvature	HOMA Value	LOLIPOP Value	Bird Aromaticity Index
A	2.13677 × 10^−5^	−0.015717083	0.995029	4.62163	97.174013
B	0.002116365	−0.018111547	0.961494	2.642712	87.617472
C	2.14365 × 10^−5^	−0.015807651	0.993422	5.569972	96.640669

**Table 14:** Binding affinities and molecular interactions between the designated compound and Lp-PLA2 (PDB ID: 6M07).

Ligand	Receptor	Interaction	Distance	E (kcal/mol)	Score (kcal/mol)
N 19	NE2 GLN 352 (A)	H-acceptor	3.56	−0.8	−7.05
N 36	N LEU 153 (A)	H-acceptor	2.85	−4.2	
N 36	N PHE 274 (A)	H-acceptor	3.39	−2.8	
6-ring	CZ PHE 110 (A)	pi-H	3.83	−1.3	

## Data Availability

All relevant data pertaining to this study are contained within the manuscript’s content.
